# Sublethal effects of parasitism on ruminants can have cascading consequences for ecosystems

**DOI:** 10.1073/pnas.2117381119

**Published:** 2022-05-09

**Authors:** Amanda M. Koltz, David J. Civitello, Daniel J. Becker, Sharon L. Deem, Aimée T. Classen, Brandon Barton, Maris Brenn-White, Zoë E. Johnson, Susan Kutz, Matthew Malishev, Daniel L. Preston, J. Trevor Vannatta, Rachel M. Penczykowski, Vanessa O. Ezenwa

**Affiliations:** ^a^Department of Biology, Washington University in St. Louis, St. Louis, MO 63130;; ^b^Department of Biology, Emory University, Atlanta, GA 30312;; ^c^Department of Biology, University of Oklahoma, Norman, OK 73019;; ^d^Saint Louis Zoo Institute for Conservation Medicine, Saint Louis, MO 63110;; ^e^Ecology and Evolutionary Biology Department, University of Michigan, Ann Arbor, MI 48109;; ^f^Department of Biological Sciences, University of Manitoba, Winnipeg, MB R3T 2N2, Canada;; ^g^Department of Biological Sciences, Mississippi State University, Mississippi State, MS 39762;; ^h^Department of Ecosystem and Public Health, Faculty of Veterinary Medicine, University of Calgary, Calgary, AB T2N 4N1, Canada;; ^i^Department of Fish, Wildlife and Conservation Biology, Colorado State University, Fort Collins, CO 80523;; ^j^Department of Biological Sciences, Purdue University, West Lafayette, IN 47907;; ^k^Department of Ecology and Evolutionary Biology, Yale University, New Haven, CT 06511

**Keywords:** helminth, herbivore, trophic cascade, feeding rate, producer

## Abstract

Parasitic infections are common, but how they shape ecosystem-level processes is understudied. Using a mathematical model and meta-analysis, we explored the potential for helminth parasites to trigger trophic cascades through lethal and sublethal effects imposed on herbivorous ruminant hosts after infection. First, using the model, we linked negative effects of parasitic infection on host survival, fecundity, and feeding rate to host and producer biomass. Our model, parameterized with data from a well-documented producer–caribou–helminth system, reveals that even moderate impacts of parasites on host survival, fecundity, or feeding rate can have cascading effects on ruminant host and producer biomass. Second, using meta-analysis, we investigated the links between helminth infections and traits of free-living ruminant hosts in nature. We found that helminth infections tend to exert negative but sublethal effects on ruminant hosts. Specifically, infection significantly reduces host feeding rates, body mass, and body condition but has weak and highly variable effects on survival and fecundity. Together, these findings suggest that while helminth parasites can trigger trophic cascades through multiple mechanisms, overlooked sublethal effects on nonreproductive traits likely dominate their impacts on ecosystems. In particular, by reducing ruminant herbivory, pervasive helminth infections may contribute to a greener world.

## Significance

We found that pervasive parasitic infections reduce herbivory rates and can trigger trophic cascades. Lethal parasites clearly have cascading impacts on ecosystems, but whether common sublethal infections have similar effects is largely unknown. Using a mathematical model, we probed how parasites that reduce host survival, fecundity, or feeding rates can indirectly alter producer biomass in a helminth–ruminant system. We found that both lethal and sublethal infections triggered trophic cascades by altering the biomass of ruminant herbivore hosts and their resources. However, a global meta-analysis revealed that helminths tend to have pervasive sublethal effects on free-living ruminants, including by reducing host feeding rates. Our findings suggest there are widespread, but overlooked, ecological consequences of sublethal infections in natural ecosystems.

Predators are well known to trigger trophic cascades by affecting the densities and traits of their prey (1). Emerging evidence suggests that parasites, including microparasites (bacteria, fungi, protozoa, and viruses) and macroparasites (helminths and arthropods) also trigger trophic cascades in similar ways (2, 3). Both predators and parasites can have cascading effects on ecosystems by killing their victims [i.e., consumptive effects on density (2, 4)] or by causing sublethal harm to victims prior to exploitation [i.e., nonconsumptive effects on traits (5, 6)]. However, unlike predators, parasites also modify victim traits by consuming but not killing their hosts (2). This raises the question of whether consumptive (i.e., postinfection) impacts of parasites occur primarily through lethal or sublethal effects on hosts (2, 7). Since nearly all organisms have long-lasting, intimate associations with a multitude of parasites, understanding the extent to which parasites trigger trophic cascades may be equally, if not more, important than for predators (8). A better understanding of the effects of parasites is especially critical because changing environmental conditions are currently affecting the abundance and diversity of parasitic species across the globe (9–12).

It is no surprise that lethal infections can have cascading effects on lower trophic levels and ecosystem functioning. One of the best examples of this was revealed by the eradication of rinderpest virus. In the late 19th century, rinderpest killed 80 to 90% of domestic and wild ruminants in sub-Saharan Africa. Following a successful vaccination campaign in cattle, release of wildebeest populations from infection dramatically altered carbon storage in the Serengeti ecosystem (13). However, unlike the rinderpest example, many infections are not lethal. Indeed, most animals survive countless infections caused by a range of parasites over their lifetimes. Such sublethal infections may also trigger trophic cascades through the modification of host behaviors or other phenotypic traits (7). For example, parasitic infection is associated with shifts in host body size and life history (14), movement behavior (15, 16), dietary preferences (17), nutrient processing (18, 19), vulnerability to predation (20–24), offspring sex and size (25), and both increased (26) and decreased (27–29) feeding rates. Changes in any of these host traits are likely to affect lower trophic levels, particularly if a host is abundant or plays a keystone role in the community.

Given the wide-ranging consumptive effects of parasites on hosts, parasite-initiated trophic cascades could emerge through a variety of mechanisms. Yet, the potential for parasites to indirectly affect ecosystem-level processes such as biomass production or nutrient cycling is largely missing from eco-epidemiological theory (2), with notable exceptions for primary producer and bacterial hosts (30–32). This is because epidemiological models typically treat hosts and parasites as separate from the surrounding ecosystem, thereby ignoring other important components of the food web, such as predators and resources (33–35). Existing resource–host–parasite models indicate that lethal parasites can stabilize resource–host oscillations, regulate host density, and relieve herbivory pressure on plants, the latter of which causes trophic cascades (36). Infections that alter the relative biomass at different trophic levels (15, 27) may also affect overall ecosystem stability (30, 36). Although sublethal infections are incredibly common (37), the potential for parasite-induced changes in host traits to have cascading consequences for ecosystems has rarely been explored theoretically (see ref. 29 for theory centered around microparasite infection in an aquatic invertebrate host). Likewise, there are very few empirical studies linking sublethal effects of parasites to trophic cascades, and those from terrestrial systems are almost exclusively limited to invertebrate hosts (refs. 2, 7, and 38 and references therein). It is therefore unclear if such findings would generalize to vertebrate hosts, their associated parasites, and terrestrial ecosystem-level impacts.

Helminths are important parasites of vertebrates, and the individual- and population-level effects of helminths on their hosts have been studied across a range of taxa (e.g., refs. 39–44). In this study, we focus on free-living ruminants (order Artiodactyla), because they are a globally distributed group of mammals that occur in almost every terrestrial biome. These hosts occupy central positions in terrestrial food webs as key primary consumers and prey for secondary consumers, and many have large impacts on ecosystems through herbivory and nutrient cycling via waste (45). Because of the economic importance of both domesticated and free-living ruminants, their parasites are well-described (46). Likewise, the consumptive impacts of parasites, including helminths, on ruminants have been intensively studied (47, 48). Importantly, infection by helminths can be lethal (e.g., refs. 39, 49, and 50) or sublethal, and the latter can have both reproductive and nonreproductive consequences for ruminant hosts (e.g., refs. 25, 41, and 51–53). Together, these traits make ruminants and their helminth parasites an excellent system to investigate how lethal vs. sublethal (including reproductive and nonreproductive) effects of infection scale up to affect host population dynamics and ecosystem function.

In our study, we combined mechanistic models with a comprehensive meta-analysis to investigate the potential for parasitic infections to trigger trophic cascades via their effects on a suite of key host traits. First, using mechanistic models, we tested the extent to which helminth-induced variation in the survival, fecundity, and feeding rate of herbivorous hosts affects the population dynamics and biomass of parasites, hosts, and primary producers. To explore how parasite-induced changes in these host traits might trigger trophic cascades, we parameterized our model using published data from caribou and reindeer (both *Rangifer tarandus*, henceforth referred to as caribou) and their common gastrointestinal helminth parasites, *Ostertagia* spp. (48). Caribou and their helminths are among the best-studied wild ruminant–parasite systems, in part because of the ecological, economic, and cultural importance of caribou in tundra ecosystems (54–57) and the ongoing disease threats to the system posed by climate change (48, 58). Second, we conducted an independent and comprehensive meta-analysis of the effects of helminth infection on the same traits (survival, fecundity, and feeding rate) of free-living ruminant hosts in order to evaluate empirical support for the mechanisms by which helminths are most likely to trigger trophic cascades in natural ecosystems globally. We also quantified the effects of helminth infection on host body mass and body condition, because changes in these traits could impact host survival, reproduction, and feeding rates (e.g., refs. 59–61). Our findings reveal that helminth parasitism has the potential to trigger trophic cascades via both lethal and sublethal effects on hosts but that sublethal effects are dominant in nature. As a consequence, sublethal parasitic infections may be an overlooked driver of ecosystem-level processes.

## Results

### Model Results.

**Fig. 1.**
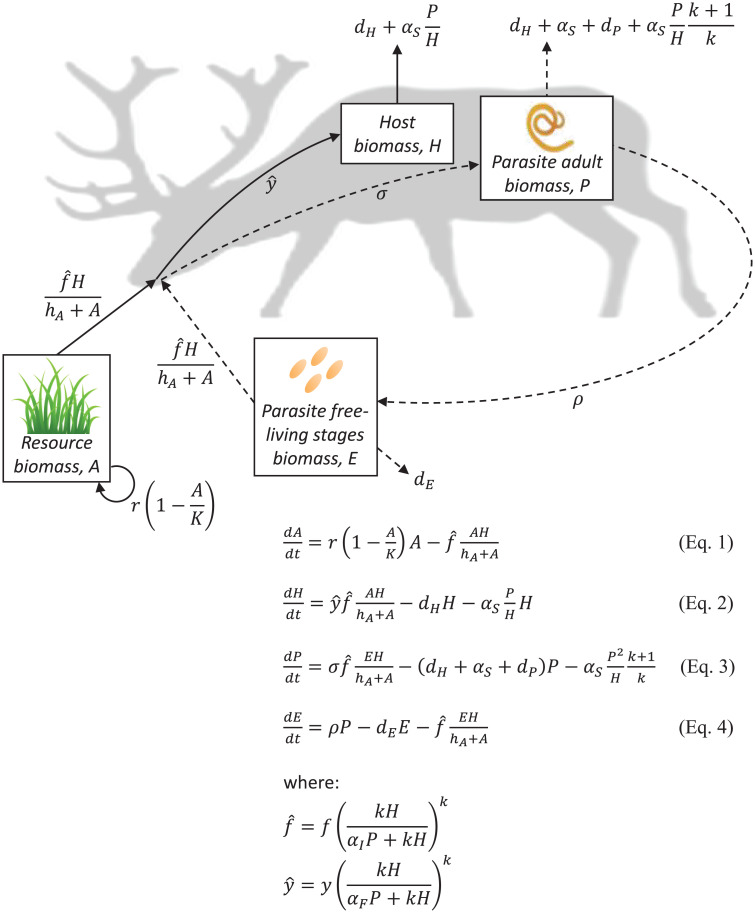
Mechanistic model framework for testing how effects of parasites on the survival (*α_S_*), fecundity (*α_F_*), and resource intake (*α_I_*) of herbivorous hosts affect the population (biomass) dynamics of parasites, free-living parasite propagules, hosts, and producers. Arrows indicate fluxes between compartments, and rates written next to arrows are per capita of the compartment where the arrow begins. Dashed lines indicate life-cycle transitions for the parasite.

To explore effects of infection in a macroparasite– herbivorous host system, we used a model that builds upon a previous framework by Smith and Grenfell (36) to track densities of producers (*A*), herbivorous hosts (*H*), adult parasites within hosts (*P*), and free-living stages of the parasite in the environment (*E*) through time using ordinary differential equations (Fig. 1). We used estimates from the literature for the genus *Ostertagia* (Table 1) to parameterize the model for caribou hosts that are commonly infected by *Ostertagia gruehneri* and that experience negative effects of these infections (51, 62). Caribou diets consist of a mix of plants and lichens. In our model, we represent these autotrophic resources with a single state variable because they are both being consumed; for simplicity, we refer to them throughout as producers. First, we used a fixed set of parameters to evaluate the stabilizing effects of parasites on the underlying consumer–resource population cycles inherent to the herbivore–autotroph submodel (i.e., the classic Rosenzweig–MacArthur predator–prey model). Second, we assessed the extent to which parasite virulence (i.e., the degree to which parasites harm the host) influences the magnitude of trophic cascades in the caribou–*Ostertagia–*producer system by varying the negative effects of the parasite on host survival (*α_S_*), fecundity (*α_F_*), and resource intake (*α_I_*). Last, we conducted a sensitivity analysis of all model parameters to assess the robustness of our inferences regarding parasite harm parameters and the general relationship between all model parameters and both the stability of the model resource–host–parasite system and trophic cascade strength.

**Table 1.** State variable names, descriptions, values, and units from mechanistic model parameterized with data from the tundra producer–caribou–helminth (*Ostertagia*) system*

State variable	Parameter	Description	Value	Ref.
Producer resources	*r*	Intrinsic growth rate	0.2 kg⋅kg^−1^⋅d^−1^	106
	*K*	Carrying capacity	200,000 kg⋅km^−2^	107
Herbivores	*f*	Foraging rate	2 kg⋅d^−1^	108
	*h_A_*	Half saturation constant	50,000 kg⋅km^−2^	107
	*d_H_*	Herbivore death rate	0.0003 kg⋅d^−1^	109
	*y*	Yield of caribou biomass from producer biomass	0.25 kg⋅kg^−1^	—
Parasites	*α_I_*	Parasite intensity-dependent effect on host resource intake	Varied, 10^−8^ to 10^−3^	—
	*α_S_*	Parasite intensity-dependent effect on host survival	Varied, 10^−8^ to 10^−3^	—
	*α_F_*	Parasite intensity-dependent effect on host fecundity	Varied, 10^−8^ to 10^−3^	—
	*ρ*	Production of free-living stages of parasite	284 eggs⋅adult female parasite^−1^⋅d^−1^	110
	*σ*	Infection probability given egg ingestion	0.3 d^−1^	110
	*d_P_*	Death rate of parasite	0.03 kg⋅d^−1^	110
	*d_E_*	Death rate of eggs	0.1 eggs⋅d^−1^	111
	*k*	Parasite aggregation factor from negative binomial distribution	2	65, 73, 74

*For yield, it is assumed that caribou gain 1 kg body weight for every 4 kg of resources consumed.

#### Effects of parasitism on host and resource dynamics are host trait dependent.

**Fig. 2.**
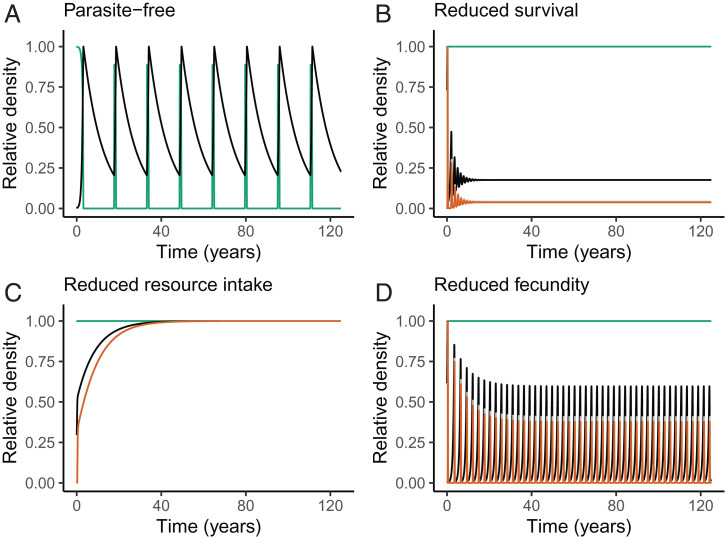
Model results of changing densities of producers (green), host herbivores (black), adult parasites (orange), and parasite propagules (gray; not visible because of adult parasites) in the environment under varying scenarios of parasite effects on hosts. Simulations shown are (*A*) without parasites, or with parasites that have negative intensity-dependent effects on (*B*) survivorship, (*C*) host resource intake, or (*D*) fecundity (note that parasite population is >0). The model was parameterized with values corresponding to the producer–caribou–*Ostertagia* system (Table 1) with herbivores, adult parasites, and free-living stages of the parasite expressed as number per square kilometer and producer biomass expressed in kilograms per square kilometer. The density values are visualized as relative to the maximum of each group in each panel.

In the absence of parasites, the model collapsed to the Rosenzweig–MacArthur model for producers and herbivores, producing stable coexistence at low producer growth rates and low herbivory rates but oscillating herbivore population sizes as producer productivity increased (63). Therefore, as expected, parasite-free simulations using parameter estimates for the *Ostertagia–*caribou system displayed limit cycles (Fig. 2*A*). Adding a parasite with only intensity-dependent negative effects on host survivorship stabilized the producer–herbivore interaction (Fig. 2*B*; *α_S_* = 10^−3^, *α_F_* = 0, *α_I_* = 0), consistent with previous results from similar models (36). Adding a parasite with only intensity-dependent effects on host resource intake also stabilized the underlying producer–herbivore interaction (Fig. 2*C*; *α_I_* = 10^−3^, *α_S_* = 0, *α_F_* = 0). Importantly, the stabilizing effects of parasite-induced declines in host resource intake occurred via a different mechanism than that of parasite effects on host survivorship (Fig. 2*B*). In this case, large parasite populations suppressed further parasite transmission by reducing host resource intake, which consequently reduced exposure of hosts to parasites. Finally, adding a parasite with only intensity-dependent effects on fecundity failed to stabilize producer–herbivore cycles, thus enabling the parasite population to grow exponentially (Fig. 2*D*; *α_F_* = 10^−3^, *α_S_* = 0, *α_I_* = 0).

**Fig. 3.**
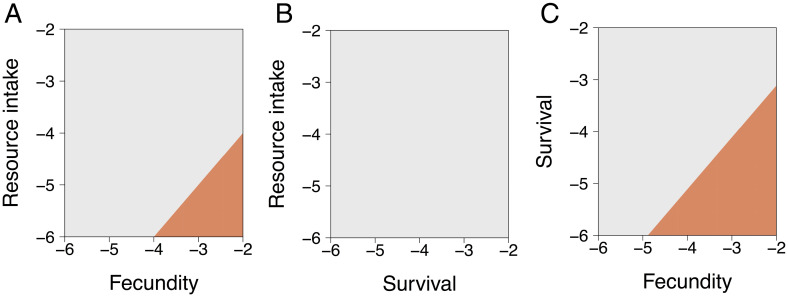
Two-parameter diagrams illustrating the qualitative behavior of the mechanistic model (Fig. 1), which oscillates in the absence of disease. Panels display variation in the magnitude of parasite virulence for pairs of lethal (survival) and sublethal (fecundity, resource intake) components included in the model (log_10_-transformed, setting the third component to zero). Parasites that harm any combination of host survival and resource intake consistently stabilize oscillations (gray; *B*). In contrast, parasites that harm host fecundity generate producer–host–parasite oscillations (orange), even in combination with a component of virulence that is otherwise stabilizing (*A* and *C*). The model is parameterized with values corresponding to the producer–caribou–*Ostertagia* system (Table 1).

Our model also demonstrates that negative effects of parasites on host traits can have qualitatively different dynamical consequences depending on the trait involved. Specifically, negative effects on host survivorship caused density-dependent increases in parasite mortality, because parasites within infected hosts are simultaneously killed with the hosts. Likewise, negative effects on host resource intake caused density-dependent decreases in parasite transmission. In both of these scenarios, stabilization occurs due to negative density dependence on the parasite. When mean infection intensity is high, average parasite mortality becomes higher and/or average exposure rate becomes lower due to the infection negatively influencing host survival or ingestion rate, respectively. In contrast, negative effects on host fecundity did not impose any density-dependent negative feedback on the parasite population and, therefore, did not stabilize the underlying producer–herbivore cycles. Interestingly, in simulations with parasite-induced negative effects on two host traits simultaneously, we found that effects on fecundity always destabilized producer–host–parasite dynamics, while negative effects of parasites on host survival or host resource intake consistently stabilized producer–host–parasite dynamics (Fig. 3). When parasite effects on fecundity were paired with one of the stabilizing effects (survival or resource intake), greater reductions in fecundity were required to generate cycles (Fig. 3 *A* and *C*).

Our global sensitivity analysis indicated that these results generally hold over a much broader range of parameter space (Latin hypercube sampling sensitivity analysis on the presence and amplitude of cycles (64); ranges: 0 to 1 for all parameters representing proportions or probabilities; 10^−8^ to 10^−3^ for parasite harm parameters *α_I_*, *α_S_*, and *α_F_*; 10-fold variation above and below estimates indicated in Table 1 for all other parameters). In addition, each mechanism of parasite-induced harm exhibited a different pattern (*SI Appendix*, Fig. S6). Parasite harm to resource intake was identified as consistently and strongly stabilizing to the producer–host–parasite system. In contrast, parasite harm to fecundity was consistently destabilizing. Finally, harm to host survival was stabilizing when parasites were aggregated [as is consistently seen for macroparasites (65)] but destabilizing when parasite aggregation was weak. Reductions in parasite aggregation among hosts also tended to destabilize the producer–host–parasite system (*SI Appendix*, Fig. S6).

#### Trophic cascades can be triggered by lethal and sublethal infections.

**Fig. 4.**
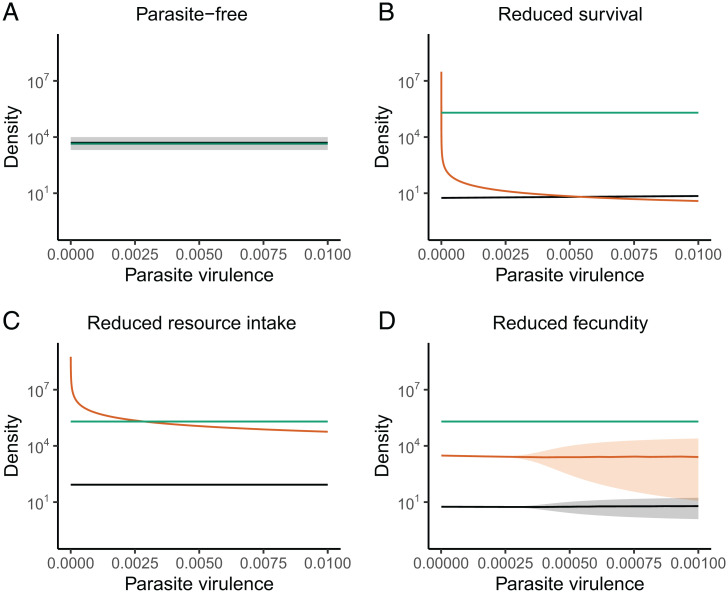
Individual effects of lethal (survival) and sublethal (resource intake, fecundity) components of parasite virulence on producers (green), host herbivores (black), and adult parasites (orange). (*A*) Parasite-free communities result in relatively high host biomass and low producer biomass compared to communities with parasites included, demonstrating top down-regulation of primary production by herbivorous hosts (shown for clarity of comparison with other panels). When considering parasite effects on individual host traits, (*B*) reduced host survivorship (*C*) reduced host resource intake, or (*D*) reduced host fecundity all trigger trophic cascades; these outcomes result in reduced host herbivore biomass and elevated producer biomass. In addition, while negative effects of infection on (*B*) host survivorship or (*C*) host resource intake stabilize resource–host oscillations, infections that reduce host fecundity (*D*) are destabilizing. Lines and shaded envelopes in *A* and *D* indicate the mean ± SE for each density, given the cycling dynamics (herbivore data obscures producer data in *A*) during the last 10,000 time steps of the model after initial transient dynamics. The model was parameterized with values corresponding to the producer–caribou–*Ostertagia* system (Table 1) with herbivores and adult parasites expressed as number per square kilometer and producer biomass expressed in kilograms per square kilometer.

Trophic cascades were triggered by lethal (survival) and both reproductive (fecundity) and nonreproductive (resource intake) sublethal effects of parasitic infection in our model. Specifically, in comparison to the parasite-free scenario (Fig. 4*A*), producer biomass was higher when parasites were present regardless of the host traits affected. Similar to classic predator-induced trophic cascades, lethal parasitic infections reduced herbivore densities and indirectly released producers from top-down regulation (Fig. 4*B*). In addition, sublethal effects that reduced rates of host resource intake (Fig. 4*C*) or fecundity (Fig. 4*D*) increased producer biomass compared to the parasite-free scenario. Interestingly, trophic cascades triggered by sublethal effects of parasites were as strong as those triggered by lethal effects. One potential explanation of this phenomenon is that, all else equal, high virulence on host survival limits the abundance of parasites, thereby limiting their population-level impacts. An identical phenomenon can be seen in the R_0_ criterion from classic susceptible–infected-type models, in which high host mortality limits the infectious period and thus total transmission potential (66).

In our global sensitivity analysis, we found that the magnitude of the trophic cascade was insensitive to the parasite harm parameters but highly sensitive to the foraging traits of the hosts (indicated by the relative change in plant biomass with vs. without the parasite; *SI Appendix*, Fig. S6). Specifically, larger trophic cascades occurred when hosts had higher feeding rates (*f*) and fecundity conversion efficiency (*y*) as well as lower background death rate (*d_H_*) and lower half-saturation constant in their type II functional response (*h_A_*). Taken together, these traits all contributed to a greater ability of hosts to suppress producer density in the absence of parasitism. Thus, larger trophic cascades can be triggered by parasites causing any type of harm if they release producers from regulation by rapidly feeding and efficient herbivore hosts [i.e., those with low R* values in the context of resource competition theory (67)].

### Meta-Analysis Results.

We used a meta-analysis to quantify the effects of helminths on the same herbivore traits (survival, fecundity, and resource intake) in natural systems as those shown to trigger trophic cascades in our model. To do this, we conducted a systematic literature search for studies that measured the effects of helminths on these three traits, in addition to body mass and body condition, in free-living and/or wild ruminants. After excluding studies which did not meet our inclusion criteria (*SI Appendix*, Fig. S1), our final dataset included 259 records from 59 studies spanning 18 host species (*SI Appendix*, Fig. S2) and five global regions (*SI Appendix*, Fig. S3).

#### Helminth effects on free-living ruminant hosts tend to be sublethal.

**Fig. 5.**
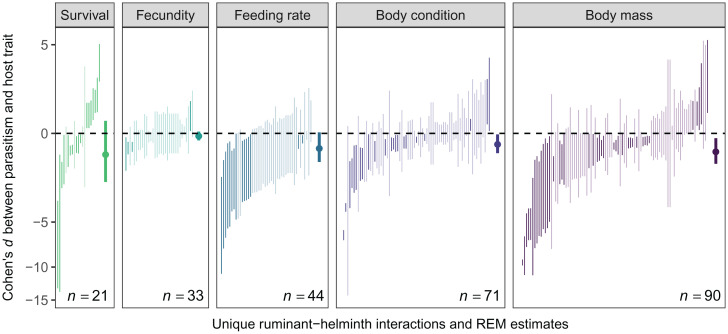
Meta-analysis results on associations between helminth infection and the survival, fecundity, feeding rate, body condition, and body mass of free-living ruminant hosts. Lines represent the 95% CI for individual effect sizes (Cohen’s *d*), shaded by whether they cross zero, and the *n* in each panel refers to the number of records per trait (total *n =* 259). Filled circles display the mean estimates and 95% CIs for each random-effects model; vertical axes are displayed with a modulus transformation to accommodate skewed effect size distributions.

We observed significant heterogeneity across all effect sizes (*I*^2^ = 0.97, *Q_258_* = 14,134, *P* < 0.001), defined as the standard mean difference in each trait between infected and uninfected hosts. However, on average, helminth infection significantly reduced host performance as defined by the focal host traits (*d* = –0.47, 95% CI = –0.77 to –0.18, *P* = 0.001), especially for experimental studies (*d* = –0.65) compared to observational studies (*d* = –0.38; *Q_1_* = 3.89, *P* = 0.05, *R*^2^ = 0.03). Examining each host trait individually, we found that helminth infection significantly reduced body mass (*d* = –0.61, 95% CI = –1.11 to –0.14) and body condition (*d* = –0.34, 95% CI = –0.66 to –0.03), and we found weaker but still generally negative effects of infection on feeding rate (*d* = –0.48, 95% CI = –1.03 to 0.03; Fig. 5 and *SI Appendix*, Table S2). Helminth infection was not significantly associated with either fecundity (*d* = –0.08, 95% CI = –0.21 to 0.05) or survival (*d* = –0.72, 95% CI = –2.05 to 0.40), although estimated mean effect sizes were both negative, and effect sizes for survival were highly variable (*I*^2^ = 0.99). Additionally, across all host traits, the majority of records had effect sizes that were negative in direction (mass: 78%, body condition: 66%, feeding rate: 95%, fecundity: 82%, survival: 62%). The direction of these mean effect size estimates was generally consistent when we stratified this analysis by study type (experimental vs. observational), although sample sizes were small for some comparisons. When considering experimental data alone (*SI Appendix*, Fig. S4 and Table S2), infection significantly reduced body mass and body condition; both feeding rate and fecundity were weakly negatively affected by infection as well. When considering just observational data (*SI Appendix*, Fig. S4 and Table S2), only fecundity and body mass were significantly and negatively associated with helminth infection. For those records that included caribou as the focal host (*n =* 71 records from 10 studies; *SI Appendix*, Fig. S5 and Table S2), helminth infection was negatively associated with body condition (*d* = –0.23, 95% CI = –0.43 to –0.03) and feeding rate (*d* = –0.87, 95% CI = –1.10 to –0.64), supporting the use of the caribou system as a case study for our modeling exercise.

## Discussion

By combining a mechanistic model and meta-analysis, we found that both lethal and sublethal effects of helminth parasitism can modify interactions between herbivores and their resources but that sublethal effects occur more commonly in nature. First, our model showed that parasites that affect host survival or feeding rate stabilized producer–herbivore population cycles (Figs. 2 *B* and *C* and 3*B*), whereas parasites that affect host fecundity were more likely to have destabilizing effects (Figs. 2*D* and 3 *A* and *C*). Second, similar to predators, lethal effects of parasites (i.e., reductions in survival) triggered classic trophic cascades (2, 68) via a reduction in herbivore density and concomitant release of producers from top-down control (Fig. 4*B*). Third, sublethal effects of infection—including reduced host feeding rates (Fig. 4*C*) and reduced fecundity (Fig. 4*D*)—also caused trophic cascades, with effects on primary producer biomass that were as strong as those caused by reductions in herbivore host survival (Fig. 4*B*). Whereas parasites that reduced herbivore host resource intake triggered trophic cascades through effects on a host trait (i.e., feeding rate), parasites that affected host fecundity and survival triggered cascades through effects on host density (i.e., by reducing the number of herbivores on the landscape). Finally, while our model revealed that helminth parasites can trigger trophic cascades via diverse mechanisms, the results of our meta-analysis indicated that the actual effects of helminth infections on free-living ruminant herbivores are predominantly sublethal (Fig. 5). Given that free-living ruminants play keystone roles in many ecosystems (69–71) and that helminth parasites are ubiquitous, these sublethal effects (e.g., reduced feeding rates) could have broad-scale ecosystem consequences. Taken together, our findings support a role for sublethal parasitic infections as a global driver of ecosystem dynamics through the modification of host functional traits that ultimately impact producer biomass.

Interestingly, when varying the intensity of multiple parasite effects on hosts simultaneously, we found that certain modes of parasite harm can stabilize host–resource oscillations, whereas others may destabilize host–resource interactions (Fig. 3 and *SI Appendix*, Fig. S6). The stabilizing effects of harm to host feeding rate that we observed for the *Ostertagia*–caribou system stand in contrast to the destabilizing effects of harm to fecundity both in this system and in models of helminth infections in red grouse populations (72). Our model indicates that these alternative stabilizing and destabilizing outcomes arise via different density-dependent mechanisms. Specifically, while negative effects of parasites on host fecundity do not directly impose any density-dependent negative feedback on the parasite population, infection-induced reduction of host feeding rate does cause density-dependent decreases in parasite transmission because hosts are exposed to parasites through feeding. We also found parasite harm to host survival can be stabilizing, but only when parasites are highly aggregated. High levels of parasite aggregation have similarly been found to promote stability in models of red grouse–nematode dynamics (72). Aggregation is typical for macroparasites (65), including for *Ostertagia* in caribou (73, 74). Consequently, a mechanistic understanding of how parasite aggregation interacts with different modes of harm to host traits is critical for predicting the stability of different resource–host–parasite systems. Notably, irrespective of whether effects of parasites on host–resource interactions were stabilizing or destabilizing, trophic cascades were possible.

Indeed, our mechanistic model indicates that trophic cascades caused by helminths may occur much more commonly than previously recognized. Specifically, both lethal and sublethal effects of infection readily generated trophic cascades across a gradient from low to high virulence. For parasites that reduced host resource intake, the strength of the trophic cascade was consistent across the virulence gradient. However, increased negative effects of parasites on host survival resulted in a very slightly weakened trophic cascade on producer biomass (not detectable in Fig. 4*B*) due to an increase in host density. This pattern of increased host density occurred in part because higher host mortality shortened the infectious period, resulting in reduced parasite population densities (75). Overall, however, we found that there was little sensitivity to the magnitude of virulence for any of the three host traits investigated. By contrast, in another recent modeling study, equilibrium biomass of plant hosts declined with increasing virulence of a pathogen (30). Fungal parasites of zooplankton hosts can also trigger trophic cascades or lead to increased host density, depending on resource productivity and the relative harm of infection to host feeding rate versus survival (29). These divergent outcomes highlight the need for future work delineating how parasite effects on ecosystems vary according to the type of parasite (e.g., macro- vs. microparasites), host trophic level, and type of harm to hosts. Interestingly, our results contrast with previous work suggesting that for macroparasites aggregation reduces their potential to trigger trophic cascades (2). Instead, we find that these common parasites have broad potential to induce ecosystem-level effects.

Despite the potential for helminths to alter community dynamics through multiple mechanisms, nonreproductive sublethal effects appear to be the most common way in which helminths trigger trophic cascades through ruminant hosts in nature. Our meta-analysis indicated that helminth infection consistently reduced host feeding rate, body condition, and body mass but on average did not affect fecundity or survival. Persistent negative effects of helminths on host body condition and mass may reflect reduced resource intake by infected individuals, but downstream effects on demographic traits such as host fecundity or survival may be weaker or more difficult to detect. Overall, however, these results support the expectation that helminth infections are harmful to host health but that effects detected in free-living ruminants are predominantly sublethal (76). To date, studies on parasite-mediated trophic cascades have focused on the lethal effects of viruses and other microparasites and the sublethal, reproductive effects of macroparasitic castrators (2, 3, 7). Our findings show that noncastrating macroparasites can also trigger trophic cascades through nonreproductive consumptive effects on hosts (2). Given that nonreproductive sublethal consequences of helminth infection are common in many host–parasite systems, our findings highlight the need for further research on the community- and ecosystem-level consequences of diverse parasite effects on host function. In particular, parasite-induced changes in host feeding behavior, which have been documented across a range of host and parasite taxa (60), could have widespread ecosystem impacts. It is important to note, however, that although helminth infection was not associated with fecundity or survival on average in our meta-analysis, variation in effect sizes was extremely high for survival; in fact, several studies showed negative effects of parasitism on survival. The majority of all effect sizes per trait also showed negative signs regardless of statistical significance. Our mechanistic model suggests that even small negative effects of infection on feeding, fecundity, or survival can have important consequences for host population dynamics and producer biomass. Thus, in contexts where helminth infection reduces host survival or fecundity by even a small amount, as observed in a large fraction of studies, there may still be cascading effects on ecosystems via shifts in producer biomass production.

The findings from this study suggest that there are global impacts of sublethal parasitic infections on ecosystems. Large herbivores often play an outsized role in ecosystem function (69, 70, 77), especially in ecosystems with few trophic levels (54, 78, 79); thus, even subtle parasite impacts on herbivore traits may have cumulative cascading impacts on ecosystems. Given that helminth parasites are nearly ubiquitous within free-living populations of ruminants (44, 46, 80), and that our meta-analysis of helminth parasitism in ruminants was global in its coverage, we suggest that global herbivory rates by ruminants are likely lower as a consequence of pervasive helminth infection. If so, the world may be greener due to the top-down effects of parasites on herbivore hosts. For example, for studies in our meta-analysis in which caribou were the host species, helminth infections caused significant reductions in host feeding rates, body mass, and body condition (*SI Appendix*, Fig. S5). Furthermore, our model showed that producer biomass increased when caribou were infected with *Ostertagia,* a common gastrointestinal parasite in this species (48). Thus, in systems like the Arctic, parasites may play a significant and underappreciated role in shaping producer dynamics. Experimental work linking hosts, parasites, and producers will be required to understand if the cascading effects of parasites on ecosystems are similar in magnitude to those induced by some predator–prey interactions (e.g., refs. 1 and 81). Future work would benefit from the development of stoichiometrically explicit models [e.g., as recently developed for plant pathogens by (30) and predator-prey systems by (82)] and collection of relevant data to clarify how sublethal effects of parasites on herbivorous hosts alter fluxes of carbon and elemental nutrients through ecosystems and how such impacts vary across ecosystems and biomes.

## Conclusion

The potential for parasites to impact ecosystems, and vice versa, has been a topic of interest in recent efforts to link the fields of disease ecology and ecosystem ecology (2, 3, 7, 30, 38, 83, 84). One of the major limitations in predicting parasite effects on ecosystems is that while studies of how parasites influence host individuals or populations have been common, syntheses of parasite effects on specific host traits that can be linked to ecosystem-level processes are rare. By using a mechanistic model to connect individual-level effects of infection to population and community dynamics and a meta-analysis to evaluate empirical support for these trait-based patterns in nature, our approach furthers our understanding of the epidemiological and ecosystem consequences of common parasitic infections. Characterizing the broader ecological roles of parasites is increasingly important, because their effects on hosts in both natural and agricultural systems are in flux. For example, parasite abundance among some of the world’s most common livestock animals is increasing (85, 86), while similar parasites in wildlife are facing extinction within the next half century (9). Our findings reveal that such changes in parasite abundance and distributions may have widespread cascading impacts on ecosystems.

## Materials and Methods

### Mechanistic Model.

We tested the potential of parasites to trigger trophic cascades by using a mechanistic model of macroparasite (helminth) infections in an herbivorous host that builds on previous work (36). We used ordinary differential equations to track the densities of autotrophic producers (*A*), host/herbivores (*H*), adult parasites within hosts (*P*), and free-living stages of the parasite in the environment (*E*) through time (Fig. 1). We illustrated several key features of the model and obtained general results by parameterizing the model with data from a plant/lichen–caribou–parasite (*Ostertagia*) system in the arctic tundra (Table 1).

[1] $$\frac{dA}{dt}=r\left(1-\frac{A}{K}\right)A-f{\left(\frac{kH}{\alpha_{I}P\;+kH}\right)}^{k}\frac{AH}{h_{A}+A}$$

[2] $$\frac{dH}{dt}=y{\left(\frac{kH}{\alpha_{F}P\;+kH}\right)}^{k}f{\left(\frac{kH}{\alpha_{I}P\;+kH}\right)}^{k}\frac{AH}{h_{A}+A}-d_{H}H-\alpha_{S}P$$

[3] $$\frac{dP}{dt}=\sigma f{\left(\frac{kH}{\alpha_{I}P\;+kH}\right)}^{k}\frac{EH}{h_{A}+A}-\left(d_{H}+\alpha_{S}+d_{P}\right)P-\alpha_{S}\frac{P^{2}}{H}\frac{k+1}{k}$$

[4] $$\frac{dE}{dt}=\rho P-d_{E}E-f{\left(\frac{kH}{\alpha_{I}P\;+kH}\right)}^{k}\frac{EH}{h_{A}+A}$$

Producers grow logistically, with maximum growth rate, *r*, and carrying capacity, *K*, reflecting competition for light or nutrients. Herbivores consume producers with a type-II functional response with maximum foraging rate, *f*, and half saturation constant, *h_A_*. Parasites can exert intensity-dependent negative effects on host resource intake at cost α*_I_* (Eq. **1**). Herbivorous hosts reproduce at a total rate that depends on ingested resources multiplied by their fecundity yield, *y*, such that reduced resource intake due to parasitism has a downstream negative effect on net fecundity of hosts. Parasites can also harm fecundity yield with intensity-dependent effects at cost *α_F_*. Hosts die with a background death rate, *d_H_*, and also due to intensity-dependent negative effects of the parasite on survival, *α_S_* (Eq. **2**). Adult parasites in hosts, *P*, increase due to infection, which occurs with per-parasite infection probability, *σ*, following ingestion of free-living parasite propagules (inclusive of eggs and larvae), *E*, and is linked to the host’s type-II functional response to producers. Adult parasites are lost due to their own background mortality, *d_P_*, and when hosts die naturally or due to virulent effects on survival (Eq. **3**). To avoid the possibility of negative rates of resource intake or biomass yields, which are biologically impossible, we represent these effects as relative decreases in these trait values per parasite; thus, the *α_F_* and *α_I_* terms represent the relative decrease in these two processes as infection intensity increases. We followed ref. 87 to integrate this assumption of relative decrease with the negative binomial distribution of parasites among hosts to generate terms representing the total ingestion and fecundity across the host population (terms raised to the exponent *k* in Eqs. **1**–**4**). The final term in Eq. **3** represents the effect of parasite aggregation on the loss of parasites when heavily parasitized hosts die. Parasite propagules in the environment are produced at rate *ρ* by adult parasites, die at background rate *d_E_*, and are removed via ingestion by hosts during the transmission process. We explored the effects of parasites on the dynamics of this producer–herbivore system by solving the model numerically using the deSolve package in R (88, 89). We explored variation in each parasite-induced effect on hosts (survival [*α_S_*], resource intake [*α_I_*], and fecundity [*α_F_*]), first individually (while setting the others to zero) and then in all pairwise combinations. We ran each simulation for 40,000 daily time steps (∼110 y); after allowing for initial transient dynamics, we evaluated long-term dynamics (i.e., stable equilibrium values or the mean and SE of each state variable if the system cycled) using the last 10,000 time steps.

We conducted a global sensitivity analysis of the model to further evaluate the generality of our inferences regarding parasite effects and stability. First, we focused on two endpoints, characterizing either the presence of cycling (0 = no, 1 = yes) or the magnitude of cycling, using the coefficient of variation of host density. For the third endpoint, we focused on the magnitude of the parasite-induced trophic cascade, with the endpoint represented as proportional change in plant abundance with and without the parasite. We used a Latin hypercube sampling design with the randomLHS() function from the lhs R package (64). We drew 60,000 randomly generated parameter sets across broad ranges of parameters and then simulated the model as above. Parameter ranges for this sensitivity analysis were 0 to 1 for all parameters representing proportions or probabilities and 10^−8^ to 10^−3^ for parasite harm parameters *α_I_*, *α_S_*, and *α_F_*. All other parameters varied 10-fold above and below the estimates indicated in Table 1. We sampled the parasite harm parameters uniformly over log-transformed space to more evenly sample the 10^5^-fold gradients. We then excluded any parameter sets in which the parasites failed to invade the population (∼12% of random parameter sets), because in these cases parasite traits would be irrelevant. We then computed partial rank correlation coefficients (PRCC) for all model parameters with respect to both measures of cycling. PRCC values span from −1 to 1 and can characterize monotonic relationships between model input parameters and model outputs without assuming linearity (90). In this case, larger values of either model output indicate cycling (destabilization), and therefore a negative PRCC value for a parameter represents evidence that increases in that parameter contribute to stabilizing the resource–host–parasite dynamics whereas a positive PRCC value indicates that increasing that parameter destabilizes the system. Datasets S5 and S6 contain the R code for the mechanistic model and sensitivity analysis.

### Meta-Analysis.

#### Literature search.

We performed systematic searches in BIOSIS, Science Citation Index, Zoological Record, and PubMed for papers published between 1 January 1980 and 24 February 2019 (the date of searches) using search strings restricted to free-living ruminant–helminth interactions (*SI Appendix*). We retrieved a total of 4,371 records, 2,217 of which were unique records after removing duplicates based on matching title, abstract, and/or DOI. We screened the titles and abstracts of all 2,217 unique records using metagear (91) in R and excluded studies that did not pertain to free-living ruminant–helminth interactions (i.e., all domestic or livestock hosts were excluded) or that did not include data on any of the focal host variables (i.e., survival, fecundity, feeding rate, body mass, or condition). We retained 390 studies after this first screening phase and assessed the full text of each of these articles for eligibility. In several cases where we determined that a study was potentially eligible but lacked necessary information for inclusion, we contacted the authors directly to request clarification. Ultimately, 59 articles contained sufficient data and methodological details for inclusion in the meta-analysis (*SI Appendix* and *SI Appendix*, Fig. S1).

#### Data extraction.

From each study, we recorded the ruminant species, helminth species (or broader taxon), response variable (i.e., body mass, body condition, fecundity, feeding rate, survival), sample size, test statistics for the relationship between parasitism and the response, and the direction of effect (i.e., positive or negative) between parasitism and the response. We also recorded study attributes including location, type (i.e., experimental or observational), and duration.

We reported effect size as the correlation-based *r* between parasitism and host traits (92). Many studies did not directly report correlation coefficients, so we first converted other test statistics (e.g., *χ*^2^, *F*, *t*, *z*, odds ratios) into *r* (93, 94). When test statistics and effect directionality were not reported, we derived them using summary data (e.g., calculating Cohen’s *d* based on means and SDs or odds ratios from tabulated counts) or converted the *P* value to a standard normal deviate *Z*-score and used the sample size to obtain *r*. When SDs were not reported, we estimated these using ranges or 95% confidence intervals (95, 96). When dispersion measures were unavailable, we imputed missing SDs using the weighted mean per study or across all data (when a study was missing one variance estimate). When exact *P* values were not reported (e.g., *P* < 0.05), we followed guidelines to convert *p* to *Z* (92). We assigned negative values to *r* when host traits were lower in infected animals. Using the metafor package, we converted all directional *r* into Fisher’s *Z* (*Z_r_*) (97). Records were excluded if these data were not reported or could not be derived or if sample sizes were less than four. The data for all records included in the meta-analysis are available in *SI Appendix*.

#### Meta-analysis.

To assess the overall relationship between helminth infection and ruminant traits, we used hierarchical meta-analysis models with the metafor package (97, 98). We nested observations within a study-level random effect to account for unit-level variance and pseudoreplication, as most studies had multiple effect sizes (34/59). We compared a series of additional random effects in order to account for repeated measures in a subset of studies (6/59), phylogenetic dependence of host species (studies did not consistently report helminth species to allow a random effect for parasite phylogeny), and spatial autocorrelation, with restricted maximum likelihood (99–102). A structure with only observation, study, and an autoregressive structure was the most parsimonious, although all possible random effects generated similar estimates of model coefficients (*SI Appendix*, Table S1). In particular, we did not find additional support for phylogenetic random effects, which suggests ruminant phylogeny does not have strong impacts on effect size and that relationships between helminths and host traits may be relatively homogenous across ruminants. All models used the rma.mv () function, weighting by sampling variance, and were fit with restricted maximum likelihood (REML). In all analyses, we back-transformed *Z_r_* to *r* and then to *d* for interpretability (i.e., the standardized mean trait difference between infected and uninfected hosts).

To assess heterogeneity among all effect sizes, we fit a random-effects model (REM; intercept only). We used the estimates of the variance components from this REM to quantify *I*^2^, the contribution of true heterogeneity to the total variance in effect size (103). We used Cochran’s *Q* to test if such heterogeneity was greater than that expected by sampling error alone (93). Next, to assess whether effect sizes varied between experimental and observational studies (104), we fit a mixed-effects model with the same random effects and study type as a moderator. We assessed moderator significance using the *Q* test (97) and derived a pseudo*R*^2^ as the proportional reduction in the summed variance components compared with those of the equivalent REM (105).

To estimate the mean relationship between helminth infection and each host trait, we repeated the above analysis by fitting a REM with the same random effects structure to each host trait subset of our data. Because the sample size for several of these traits was relatively low, we a priori stratified these analyses by observational and experimental study to assess whether stronger effects of parasitism on each trait were detected using experimental approaches (104).

## Supplementary Material

Supplementary File

Supplementary File

Supplementary File

## Data Availability

All study data are included in the article and/or supporting information.

## References

[1] W. J. Ripple , Status and ecological effects of the world’s largest carnivores. Science 343, 1241484 (2014). 24408439 10.1126/science.1241484

[2] J. C. Buck, W. J. Ripple, Infectious agents trigger trophic cascades. Trends Ecol. Evol. 32, 681–694 (2017). 28736043 10.1016/j.tree.2017.06.009

[3] D. L. Preston, J. A. Mischler, A. R. Townsend, P. T. J. Johnson, Disease ecology meets ecosystem science. Ecosystems (N. Y.) 19, 737–748 (2016).

[4] O. J. Schmitz, P. A. Hambäck, A. P. Beckerman, Trophic cascades in terrestrial systems: A review of the effects of carnivore removals on plants. Am. Nat. 155, 141–153 (2000). 10686157 10.1086/303311

[5] J. P. Suraci, M. Clinchy, L. M. Dill, D. Roberts, L. Y. Zanette, Fear of large carnivores causes a trophic cascade. Nat. Commun. 7, 10698 (2016). 26906881 10.1038/ncomms10698 PMC4766389

[6] S. B. Weinstein, J. C. Buck, H. S. Young, A landscape of disgust. Science 359, 1213–1214 (2018). 29590062 10.1126/science.aas8694

[7] J. C. Buck, Indirect effects explain the role of parasites in ecosystems. Trends Parasitol. 35, 835–847 (2019). 31444059 10.1016/j.pt.2019.07.007

[8] A. Dobson, K. D. Lafferty, A. M. Kuris, R. F. Hechinger, W. Jetz, Colloquium paper: Homage to Linnaeus: How many parasites? How many hosts? Proc. Natl. Acad. Sci. U.S.A. 105 (suppl. 1), 11482–11489 (2008). 18695218 10.1073/pnas.0803232105 PMC2556407

[9] C. J. Carlson , Parasite biodiversity faces extinction and redistribution in a changing climate. Sci. Adv. 3, e1602422 (2017). 28913417 10.1126/sciadv.1602422 PMC5587099

[10] R. R. Dunn, N. C. Harris, R. K. Colwell, L. P. Koh, N. S. Sodhi, The sixth mass coextinction: Are most endangered species parasites and mutualists? Proc. R. Soc. B-Biol. Sci. 276, 3037–3045 (2009). 10.1098/rspb.2009.0413 PMC2817118 19474041

[11] A. Gómez, E. Nichols, Neglected wild life: Parasitic biodiversity as a conservation target. Int. J. Parasitol. Parasites Wildl. 2, 222–227 (2013). 24533340 10.1016/j.ijppaw.2013.07.002 PMC3862516

[12] P. Kafle , Range expansion of muskox lungworms track rapid arctic warming: Implications for geographic colonization under climate forcing. Sci. Rep. 10, 17323 (2020). 33057173 10.1038/s41598-020-74358-5 PMC7560617

[13] R. M. Holdo , A disease-mediated trophic cascade in the Serengeti and its implications for ecosystem C. PLoS Biol. 7, e1000210 (2009). 19787022 10.1371/journal.pbio.1000210 PMC2740867

[14] D. M. Tompkins, A. M. Dunn, M. J. Smith, S. Telfer, Wildlife diseases: From individuals to ecosystems. J. Anim. Ecol. 80, 19–38 (2011). 20735792 10.1111/j.1365-2656.2010.01742.x

[15] K. N. Mouritsen, R. Poulin, Parasites boosts biodiversity and changes animal community structure by trait‐mediated indirect effects. Oikos 108, 344–350 (2005).

[16] K. O’Dwyer, T. Kamiya, R. Poulin, Altered microhabitat use and movement of littorinid gastropods: The effects of parasites. Mar. Biol. 161, 437–445 (2014).

[17] T. Lefèvre, L. Oliver, M. D. Hunter, J. C. De Roode, Evidence for trans-generational medication in nature. Ecol. Lett. 13, 1485–1493 (2010). 21040353 10.1111/j.1461-0248.2010.01537.x

[18] C. F. Narr, P. C. Frost, Does infection tilt the scales? Disease effects on the mass balance of an invertebrate nutrient recycler. Oecologia 179, 969–979 (2015). 26298190 10.1007/s00442-015-3412-5

[19] J. C. Munger, W. H. Karasov, Sublethal parasites and host energy budgets: Tapeworm infection in white‐footed mice. Ecology 70, 904–921 (1989).

[20] V. Alzaga , Body condition and parasite intensity correlates with escape capacity in Iberian hares (*Lepus granatensis*). Behav. Ecol. Sociobiol. 62, 769–775 (2008).

[21] T. Sato , Nematomorph parasites drive energy flow through a riparian ecosystem. Ecology 92, 201–207 (2011). 21560690 10.1890/09-1565.1

[22] A. R. Ives, D. L. Murray, Can sublethal parasitism destabilize predator-prey population dynamics? A model of snowshoe hares, predators and parasites. J. Anim. Ecol. 66, 265–278 (1997).

[23] D. O. Joly, F. Messier, The distribution of *Echinococcus granulosus* in moose: Evidence for parasite-induced vulnerability to predation by wolves? Oecologia 140, 586–590 (2004). 15232731 10.1007/s00442-004-1633-0

[24] S. J. Peacock, M. Krkošek, A. W. Bateman, M. A. Lewis, Parasitism and food web dynamics of juvenile Pacific salmon. Ecosphere 6, 1–16 (2015).

[25] O. A. Aleuy, E. Serrano, K. E. Ruckstuhl, E. P. Hoberg, S. Kutz, Parasite intensity drives fetal development and sex allocation in a wild ungulate. Sci. Rep. 10, 15626 (2020). 32973197 10.1038/s41598-020-72376-x PMC7518422

[26] R. J. Bernot, G. A. Lamberti, Indirect effects of a parasite on a benthic community: An experiment with trematodes, snails and periphyton. Freshw. Biol. 53, 322–329 (2008).

[27] C. L. Wood , Parasites alter community structure. Proc. Natl. Acad. Sci. U.S.A. 104, 9335–9339 (2007). 17517667 10.1073/pnas.0700062104 PMC1890495

[28] J. L. Hite, C. E. Cressler, Parasite-mediated anorexia and nutrition modulate virulence evolution. Integr. Comp. Biol. 59, 1264–1274 (2019). 31187120 10.1093/icb/icz100 PMC6863757

[29] R. M. Penczykowski , Virulent disease epidemics can increase host density by depressing foraging of hosts. Am. Nat. 199, 75–90 (2022). 34978968 10.1086/717175

[30] E. T. Borer , Elements of disease in a changing world: Modelling feedbacks between infectious disease and ecosystems. Ecol. Lett. (2020). 10.1111/ele.13617 33047456

[31] L. F. Jover, T. C. Effler, A. Buchan, S. W. Wilhelm, J. S. Weitz, The elemental composition of virus particles: Implications for marine biogeochemical cycles. Nat. Rev. Microbiol. 12, 519–528 (2014). 24931044 10.1038/nrmicro3289

[32] J. A. Fuhrman, Marine viruses and their biogeochemical and ecological effects. Nature 399, 541–548 (1999). 10376593 10.1038/21119

[33] D. J. Civitello, B. E. Allman, C. Morozumi, J. R. Rohr, Assessing the direct and indirect effects of food provisioning and nutrient enrichment on wildlife infectious disease dynamics. Philos. Trans. R. Soc. B: Biol. Sci. 373, 20170101 (2018). 10.1098/rstb.2017.0101 PMC5883004 29531153

[34] S. R. Hall , Quality matters: Resource quality for hosts and the timing of epidemics. Ecol. Lett. 12, 118–128 (2009). 19049510 10.1111/j.1461-0248.2008.01264.x

[35] K. D. Lafferty , Parasites in food webs: The ultimate missing links. Ecol. Lett. 11, 533–546 (2008). 18462196 10.1111/j.1461-0248.2008.01174.x PMC2408649

[36] G. Smith, B. T. Grenfell, Modelling of parasite populations: Gastrointestinal nematode models. Vet. Parasitol. 54, 127–143 (1994). 7846847 10.1016/0304-4017(94)90087-6

[37] P. J. Hotez , The global burden of disease study 2010: Interpretation and implications for the neglected tropical diseases. PLoS Negl. Trop. Dis. 8, e2865 (2014). 25058013 10.1371/journal.pntd.0002865 PMC4109880

[38] I. R. Fischhoff , Parasite and pathogen effects on ecosystem processes: A quantitative review. Ecosphere 11, e03057 (2020).

[39] F. M. Gulland, The role of nematode parasites in Soay sheep (*Ovis aries* L.) mortality during a population crash. Parasitology 105, 493–503 (1992). 1461688 10.1017/s0031182000074679

[40] P. J. Hudson, A. P. Dobson, D. Newborn, Prevention of population cycles by parasite removal. Science 282, 2256–2258 (1998). 9856948 10.1126/science.282.5397.2256

[41] S. D. Albon , The role of parasites in the dynamics of a reindeer population. Proc. Biol. Sci. 269, 1625–1632 (2002). 12184833 10.1098/rspb.2002.2064 PMC1691070

[42] A. B. Pedersen, T. J. Greives, The interaction of parasites and resources cause crashes in a wild mouse population. J. Anim. Ecol. 77, 370–377 (2008). 18028357 10.1111/j.1365-2656.2007.01321.x

[43] A. D. Blackwell , Helminth infection, fecundity, and age of first pregnancy in women. Science 350, 970–972 (2015). 26586763 10.1126/science.aac7902 PMC5953513

[44] R. Poulin, Parasite biodiversity revisited: Frontiers and constraints. Int. J. Parasitol. 44, 581–589 (2014). 24607559 10.1016/j.ijpara.2014.02.003

[45] W. C. Turner , Fatal attraction: Vegetation responses to nutrient inputs attract herbivores to infectious anthrax carcass sites. Proc. R. Soc. B: Biol. Sci. 281, 20141785 (2014). 10.1098/rspb.2014.1785 PMC4213624 25274365

[46] V. O. Ezenwa, S. A. Price, S. Altizer, N. D. Vitone, K. C. Cook, Host traits and parasite species richness in even and odd-toed hoofed mammals, Artiodactyla and Perissodactyla. Oikos 115, 526–536 (2006).

[47] A. R. Sykes, Parasitism and production in farm animals. Anim. Sci. 59, 155–172 (2010).

[48] S. J. Kutz , Parasites in ungulates of Arctic North America and Greenland: A view of contemporary diversity, ecology, and impact in a world under change. Adv. Parasitol. 79, 99–252 (2012). 22726643 10.1016/B978-0-12-398457-9.00002-0

[49] M. J. Farrell, T. J. Davies, Disease mortality in domesticated animals is predicted by host evolutionary relationships. Proc. Natl. Acad. Sci. U.S.A. 116, 7911–7915 (2019). 30926660 10.1073/pnas.1817323116 PMC6475420

[50] V. O. Ezenwa, A. E. Jolles, Epidemiology. Opposite effects of anthelmintic treatment on microbial infection at individual versus population scales. Science 347, 175–177 (2015). 25574023 10.1126/science.1261714

[51] R. J. Irvine, S. D. Albon, A. Stien, O. Halvorsen, A. M. Carlsson, “Manipulating parasites in an Arctic herbivore: Gastrointestinal nematodes and the population regulation of Svalbard reindeer” in Wildlife Disease Ecology: Linking Theory to Data and Application, K. Wilson, A. Fenton, D. Tomkins, Eds. (Cambridge University Press, 2020), pp. 397–426.

[52] G. Coulson, J. K. Cripps, S. Garnick, V. Bristow, I. Beveridge, Parasite insight: Assessing fitness costs, infection risks and foraging benefits relating to gastrointestinal nematodes in wild mammalian herbivores. Philos. Trans. R. Soc. B. Biol. Sci. 373, 20170197 (2018). 10.1098/rstb.2017.0197 PMC6000135 29866912

[53] O. A. Aleuy , Diversity of gastrointestinal helminths in Dall’s sheep and the negative association of the abomasal nematode, *Marshallagia marshalli*, with fitness indicators. PLoS One 13, e0192825 (2018). 29538393 10.1371/journal.pone.0192825 PMC5851548

[54] C. Bernes, K. A. Bråthen, B. C. Forbes, J. D. Speed, J. Moen, What are the impacts of reindeer/caribou (*Rangifer tarandus* L.) on arctic and alpine vegetation? A systematic review. Environ. Evid. 4, 4 (2015).

[55] R. A. Ims , “Terrestrial ecosystems” in Arctic Biodiversity Assessment: Status and Trends in Arctic Biodiversity. Conservation of Arctic Flora and Fauna (CAFF), H. Meltofte, A. B. Josefson, D. Payer, Eds. (Conservation of Arctic Flora and Fauna, 2013), pp. 384–440.

[56] K. A. Bråthen, J. Oksanen, Reindeer reduce biomass of preferred plant species. J. Veg. Sci. 12, 473–480 (2001).

[57] J. A. Fall, Regional patterns of fish and wildlife harvests in contemporary Alaska. Arctic 69, 47–64 (2016).

[58] S. J. Kutz , The Arctic as a model for anticipating, preventing, and mitigating climate change impacts on host-parasite interactions. Vet. Parasitol. 163, 217–228 (2009). 19560274 10.1016/j.vetpar.2009.06.008

[59] A. Gunn, R. J. Irvine, Subclinical parasitism and ruminant foraging strategies: A review. Wildl. Soc. Bull. 31, 117–126 (2003).

[60] J. L. Hite, A. C. Pfenning, C. E. Cressler, Starving the enemy? Feeding behavior shapes host-parasite interactions. Trends Ecol. Evol. 35, 68–80 (2020). 31604593 10.1016/j.tree.2019.08.004

[61] R. Irvine, Parasites and the dynamics of wild mammal populations. Anim. Sci. 82, 775–781 (2006).

[62] A. Stien , The impact of gastrointestinal nematodes on wild reindeer: Experimental and cross‐sectional studies. J. Anim. Ecol. 71, 937–945 (2002).

[63] M. L. Rosenzweig, R. H. MacArthur, Graphical representation and stability conditions of predator-prey interactions. Am. Nat. 97, 209–223 (1963).

[64] R. Carnell, lhs: Latin Hypercube samples. R package version 1.1.1. (2020). https://cran.r-project.org/web/packages/lhs/index.html. Accessed 20 May 2021.

[65] D. J. Shaw, A. P. Dobson, Patterns of macroparasite abundance and aggregation in wildlife populations: A quantitative review. Parasitology 111 (suppl.), S111–S127 (1995). 8632918 10.1017/s0031182000075855

[66] R. M. Anderson, R. M. May, The invasion, persistence and spread of infectious diseases within animal and plant communities. Philos. Trans. R. Soc. Lond. B Biol. Sci. 314, 533–570 (1986). 2880354 10.1098/rstb.1986.0072

[67] D. Tilman, Resources: A graphical-mechanistic approach to competition and predation. Am. Nat. 116, 362–393 (1980).

[68] S. R. Carpenter, J. F. Kitchell, The Trophic Cascade in Lakes (Cambridge University Press, 1996).

[69] N. T. Hobbs, Modification of ecosystems by ungulates. J. Wildl. Manage. 60, 695–713 (1996).

[70] E. S. Forbes , Synthesizing the effects of large, wild herbivore exclusion on ecosystem function. Funct. Ecol. 33, 1597–1610 (2019).

[71] D. M. Waller, W. S. Alverson, The white-tailed deer: A keystone herbivore. Wildlife Soc. Bull. (1973-2006) 25, 217–226.

[72] A. P. Dobson, P. J. Hudson, Regulation and stability of a free-living host-parasite system: *Trichostrongylus tenuis* in red grouse. II. Population models. J. Anim. Ecol. 61, 487–498 (1992).

[73] K. Bye, Abomasal nematodes from three Norwegian wild reindeer populations. Can. J. Zool. 65, 677–680 (1987).

[74] K. Bye, O. Halvorsen, K. Nilssen, Immigration and regional distribution of abomasal nematodes of Svalbard reindeer. J. Biogeogr. 14, 451–458 (1987).

[75] M. J. Keeling, P. Rohani, Modeling Infectious Diseases in Humans and Animals (Princeton University Press, 2011).

[76] M. T. Fox, Pathophysiology of infection with gastrointestinal nematodes in domestic ruminants: Recent developments. Vet. Parasitol. 72, 285–297, discussion 297–308 (1997). 9460203 10.1016/s0304-4017(97)00102-7

[77] R. Van Der Wal, R. D. Bardgett, K. A. Harrison, A. Stien, Vertebrate herbivores and ecosystem control: Cascading effects of faeces on tundra ecosystems. Ecography 27, 242–252 (2004).

[78] B. B. Hansen, S. Henriksen, R. Aanes, B.-E. Saether, Ungulate impact on vegetation in a two-level trophic system. Polar Biol. 30, 549–558 (2007).

[79] T. Morris, M. Letnic, Removal of an apex predator initiates a trophic cascade that extends from herbivores to vegetation and the soil nutrient pool. Proc. R. Soc. B: Biol. Sci. 284, 20170111 (2017). 10.1098/rspb.2017.0111 PMC5443940 28490624

[80] E. P. Hoberg, A. A. Kocan, L. G. Rickard, “Gastrointestinal strongyles in wild ruminants” in Parasitic Diseases of Wild Mammals, W. M. Samuel, M. J. Pybus, A. Kocan, Eds. (Wiley, 2001), p. 227.

[81] O. J. Schmitz , Animals and the zoogeochemistry of the carbon cycle. Science 362, eaar3213 (2018). 30523083 10.1126/science.aar3213

[82] S. J. Leroux, O. J. Schmitz, Predator-driven elemental cycling: The impact of predation and risk effects on ecosystem stoichiometry. Ecol. Evol. 5, 4976–4988 (2015). 26640675 10.1002/ece3.1760 PMC4662303

[83] J. T. Vannatta, D. J. Minchella, Parasites and their impact on ecosystem nutrient cycling. Trends Parasitol. 34, 452–455 (2018). 29526401 10.1016/j.pt.2018.02.007

[84] R. E. Paseka , Disease-mediated ecosystem services: Pathogens, plants, and people. Trends Ecol. Evol. 35, 731–743 (2020). 32553885 10.1016/j.tree.2020.04.003

[85] R. M. Kaplan, Drug resistance in nematodes of veterinary importance: A status report. Trends Parasitol. 20, 477–481 (2004). 15363441 10.1016/j.pt.2004.08.001

[86] J. Van Dijk, N. Sargison, F. Kenyon, P. Skuce, Climate change and infectious disease: Helminthological challenges to farmed ruminants in temperate regions. Animal. 4, 377 (2010). 22443942 10.1017/S1751731109990991

[87] O. Diekmann, M. Kretzschmar, Patterns in the effects of infectious diseases on population growth. J. Math. Biol. 29, 539–570 (1991). 1895021 10.1007/BF00164051

[88] R. C. Team, R: A Language and Environment for Statistical Computing (R Foundation for Statistical Computing, 2017).

[89] K. Soetaert, T. Petzoldt, R. W. Setzer, Solving differential equations in R: Package deSolve. J. Stat. Softw. 33, 1–25 (2010).20808728

[90] J. Wu, R. Dhingra, M. Gambhir, J. V. Remais, Sensitivity analysis of infectious disease models: Methods, advances and their application. J. R. Soc. Interface 10, 20121018 (2013). 23864497 10.1098/rsif.2012.1018 PMC3730677

[91] M. J. Lajeunesse, Facilitating systematic reviews, data extraction and meta‐analysis with the metagear package for R. Methods Ecol. Evol. 7, 323–330 (2016).

[92] R. Rosenthal, M. R. DiMatteo, Meta-analysis: Recent developments in quantitative methods for literature reviews. Annu. Rev. Psychol. 52, 59–82 (2001). 11148299 10.1146/annurev.psych.52.1.59

[93] M. Borenstein, L. V. Hedges, J. P. Higgins, H. R. Rothstein, Introduction to Meta-Analysis (John Wiley & Sons, 2011).

[94] J. Koricheva, J. Gurevitch, K. Mengersen, Handbook of Meta-Analysis in Ecology and Evolution (Princeton University Press, 2013).

[95] X. Wan, W. Wang, J. Liu, T. Tong, Estimating the sample mean and standard deviation from the sample size, median, range and/or interquartile range. BMC Med. Res. Methodol. 14, 135 (2014). 25524443 10.1186/1471-2288-14-135 PMC4383202

[96] J. P. Higgins , Cochrane Handbook for Systematic Reviews of Interventions (John Wiley & Sons, 2019).

[97] W. Viechtbauer, Conducting meta-analyses in R with the metafor package. J. Stat. Softw. 36, 1–48 (2010).

[98] S. Konstantopoulos, Fixed effects and variance components estimation in three-level meta-analysis. Res. Synth. Methods 2, 61–76 (2011). 26061600 10.1002/jrsm.35

[99] N. Cressie, Statistics for Spatial Data (John Wiley & Sons, 2015).

[100] O. Cinar, S. Nakagawa, W. Viechtbauer, Phylogenetic multilevel meta-analysis: A simulation study on the importance of modelling the phylogeny. Methods Ecol. Evol. 13, 383–395 (2022).

[101] A. Zuur, E. Ieno, N. Walker, A. Saveliev, G. Smith, Mixed Effects Models and Extensions in Ecology with R (Springer Science & Business Media, New York, 2009).

[102] K. J. Ishak, R. W. Platt, L. Joseph, J. A. Hanley, J. J. Caro, Meta-analysis of longitudinal studies. Clin. Trials 4, 525–539 (2007). 17942468 10.1177/1740774507083567

[103] A. M. Senior , Heterogeneity in ecological and evolutionary meta-analyses: Its magnitude and implications. Ecology 97, 3293–3299 (2016). 27912008 10.1002/ecy.1591

[104] C. A. Sánchez , On the relationship between body condition and parasite infection in wildlife: A review and meta-analysis. Ecol. Lett. 21, 1869–1884 (2018). 30369000 10.1111/ele.13160

[105] J. A. López-López, F. Marín-Martínez, J. Sánchez-Meca, W. Van den Noortgate, W. Viechtbauer, Estimation of the predictive power of the model in mixed-effects meta-regression: A simulation study. Br. J. Math. Stat. Psychol. 67, 30–48 (2014). 23297709 10.1111/bmsp.12002

[106] H. Poorter, C. Remkes, Leaf area ratio and net assimilation rate of 24 wild species differing in relative growth rate. Oecologia 83, 553–559 (1990). 28313192 10.1007/BF00317209

[107] J. Trudell, R. G. White, The effect of forage structure and availability on food intake, biting rate, bite size and daily eating time of reindeer. J. Appl. Ecol. 18, 63–81 (1981).

[108] D. Holleman, J. Luick, R. White, Lichen intake estimates for reindeer and caribou during winter. J. Wildl. Manage. 43, 192–201 (1979).

[109] T. Skogland, Natural selection of wild reindeer life history traits by food limitation and predation. Oikos 55, 101–110 (1989).

[110] S. H. Verschave , The parasitic phase of Ostertagia ostertagi: quantification of the main life history traits through systematic review and meta-analysis. Int. J. Parasitol. 44, 1091–1104 (2014). 25229178 10.1016/j.ijpara.2014.08.006

[111] B. T. Grenfell, G. Smith, R. M. Anderson, Maximum-likelihood estimates of the mortality and migration rates of the infective larvae of Ostertagia ostertagi and Cooperia oncophora. Parasitology 92, 643–652 (1986). 3737245 10.1017/s0031182000065501

