# Supplementary material for: Sublethal effects of parasitism on ruminants can have cascading consequences for ecosystems
Source: Proc Natl Acad Sci U S A. 2022 May 9;119(20):e2117381119. doi: 10.1073/pnas.2117381119 (PMC9171767; doi:10.1073/pnas.2117381119)
Supplement: Supplementary File [file pnas.2117381119.sapp.pdf]

PNAS
www.pnas.org

Supplementary Information for

Sublethal effects of parasitism on ruminants can have cascading consequences for ecosystems

Amanda M. Koltz[1], David J. Civitello, Daniel J. Becker, Sharon L. Deem, Aimée T. Classen, Brandon Barton, Maris Brenn-White, Zoë E. Johnson, Susan Kutz, Matthew Malishev, Daniel L. Preston, J. Trevor Vannatta, Rachel M. Penczykowski, and Vanessa O. Ezenwa

[1]Corresponding author: Amanda M. Koltz
Email: akoltz@wustl.edu

This PDF file includes:

Supplementary text for Meta-analysis Methods
Figures S1 to S6
Tables S1 to S2
Legends for Datasets S1 to S3
SI References

Other supplementary materials for this manuscript include the following:

Datasets S1 to S3
R code for mechanistic model
R code for corresponding sensitivity analysis
Two C files to accompany sensitivity analysis

Supplementary Information Text

Expanded Methods for Meta-analysis: Literature Search and Data Extraction

Literature search summary

We searched for literature on non-domesticated ruminant ungulates and their helminth parasites by querying the Biosis, SCI, PubMed, and Zoological Record databases on February 24, 2019, using the following search terms and parameters.

Search terms:
(TS= (hyemoschus OR moschiola OR tragulus OR moschus OR alces OR blastocerus OR capreolus OR hippocamelus OR mazama OR odocoileus OR ozotoceros OR pudu OR rangifer OR cervus OR dama OR elaphodus OR muntiacus OR przewalskium OR rucervus OR rusa OR hydropotes OR antilocapra OR giraffa OR okapia OR aepyceros OR alcelaphus OR beatragus OR connochaetes OR damaliscus OR ammodorcas OR antidorcas OR antelope OR dorcatragus OR eudorcas OR gazella OR litocranius OR madoqua OR nanger OR neotragus OR oreotragus OR ourebia OR procapra OR raphicerus OR saiga OR bison OR boselaphus OR bubalus OR pseudoryx OR syncerus OR taurotragus OR tetracerus OR tragelaphus OR ammotragus OR budorcas OR capricornis OR hemitragus OR naemorhedus OR oreamnos OR ovibos OR pantholops OR pseudois OR rupicapra OR cephalophus OR philantomba OR sylvicapra OR addax OR hippotragus OR oryx OR kobus OR perea OR redunca) AND ALL = (helminth* OR nematode* OR cestode* OR trematode*))

((ALL = (Capra) AND ALL = (caucasica OR falconeri OR ibex OR nubiana OR pyrenaica OR sibirica OR walie) AND ALL = (helminth* OR nematode* OR cestode* OR trematode*)))

((ALL = (Ovis) AND ALL = (Soay OR ammon OR canadensis OR dalli OR nivicola) AND ALL = (helminth* OR nematode* OR cestode* OR trematode*)))

((ALL = (Bos) AND ALL = (frontalis OR grunniens OR javanicus OR sauveli OR primigenius) AND ALL = (helminth* OR nematode* OR cetsode* OR trematode*)))

((ALL = (Axis) AND ALL = (deer OR porcinus OR calamiane OR kuhlii) AND ALL = (helminth* OR nematode* OR cestode* OR trematode*)))

Search parameters:
Search fields = All
Date range = Jan 1, 1980 - Feb 24, 2019

Removal of duplicates:
Punctuation and capitalization was removed from all titles and abstracts and duplicates were removed based on matching title, abstract, and/or DOI in R (1). Remaining duplicate records were identified by manual review of titles and abstracts.

Record summary
Total records retrieved: 4371
Records removed as duplicate: 2154
Unique records: 2217

Literature screening and data extraction

Using the unique records from the literature search, we then assessed articles for inclusion in the meta-analysis in two phases (Fig. S1):

First, we screened the titles and abstracts of all 2217 unique records to determine whether the studies included primary data involving wild (not domesticated) ruminants and their helminth parasites, and quantified parasite effects on at least one of our focal host response variables. We used the *metagear* package to randomly distribute the 2217 records among seven co-authors for screening of titles and abstracts (2). A subset of records (200 total) were dual-screened, and we performed a kappa analysis to assess inter-reviewer agreement (2). For the four pairs of reviewers dual-screening 50 records each, Cohen's kappa scores were 0.72 (substantial agreement), 0.42 (moderate agreement), 0.32 (fair agreement), and 0.15 (slight agreement). Three screeners were consistently more likely to exclude than retain records (resulting in "slight", "fair", and "moderate" agreement with their co-reviewer), and all records screened by those three people were then re-screened by the two people who had been most likely to retain records. If screeners did not both agree to retain or exclude a record, then the record was retained. We excluded 1827 studies at this phase, and retained 390 studies that were scored as either "yes" or "maybe" eligible for inclusion in the meta-analysis.

Second, we downloaded the full-text articles of the 390 retained records (Fig. S1). These full texts were assessed for eligibility in the meta-analysis based on the completeness of methodological and statistical methods (e.g., information on sample size, direction of effect, etc.). We recorded effect sizes for any studies that reported them directly; for all others, we calculated effect size of infection status on the host trait of interest based on the statistical information, sample size, and direction of effect that were included in the study. If studies measured appropriate response variables but did not report the results in a format that could be used for the meta-analysis, whenever possible, we extracted raw data from relevant tables or figures that were provided in the main text or supplements and then calculated an effect size of infection on the response variable of interest. When necessary, data were extracted from figures using Plot Digitizer (v. 2.6.8; http://plotdigitizer.sourceforge.net/). In several circumstances, we contacted authors directly to request clarifying information for studies that seemed appropriate for inclusion in the meta-analysis but that lacked certain information within the publication; references that were included thanks to cooperation by authors are noted in Dataset S3. The final dataset included 259 records from 59 studies (Fig. S1; Dataset S3). Most of the included studies measured impacts of nematode (n=194 records) or trematode parasites (n=59) on hosts; a few measured effects of cestodes (n=2) or included a mix of helminth parasites (n=4). Approximately half of the records in our meta-analysis used experiments (i.e., parasite removal or additions) to assess causal effects of helminths on host traits (n=119, 46%).

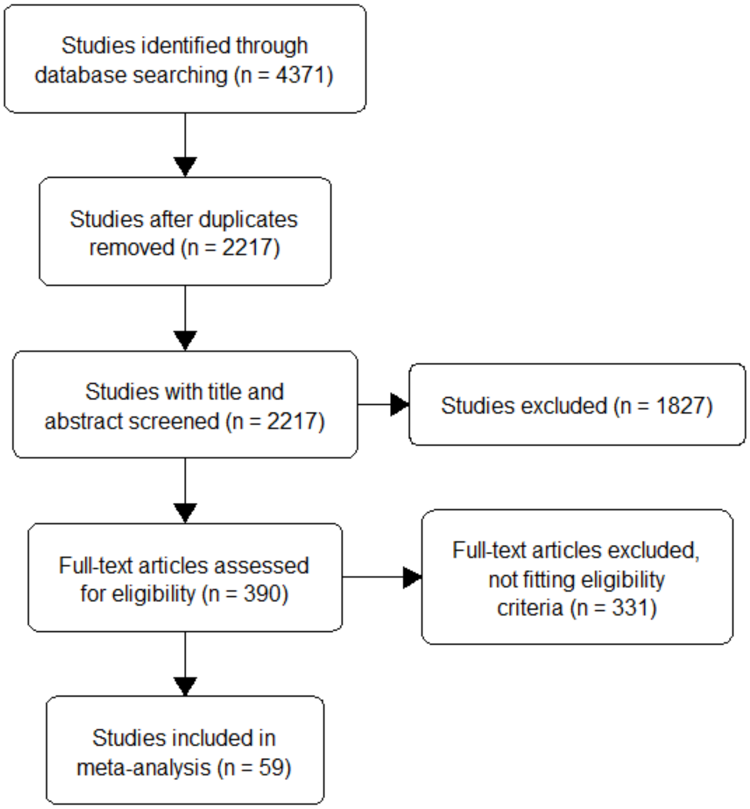

Fig. S1. The number of studies that were included at each phase of the literature screening process for the meta-analysis of helminth parasite effects on free-living ruminants. Figure visualized using PRISMA (3).

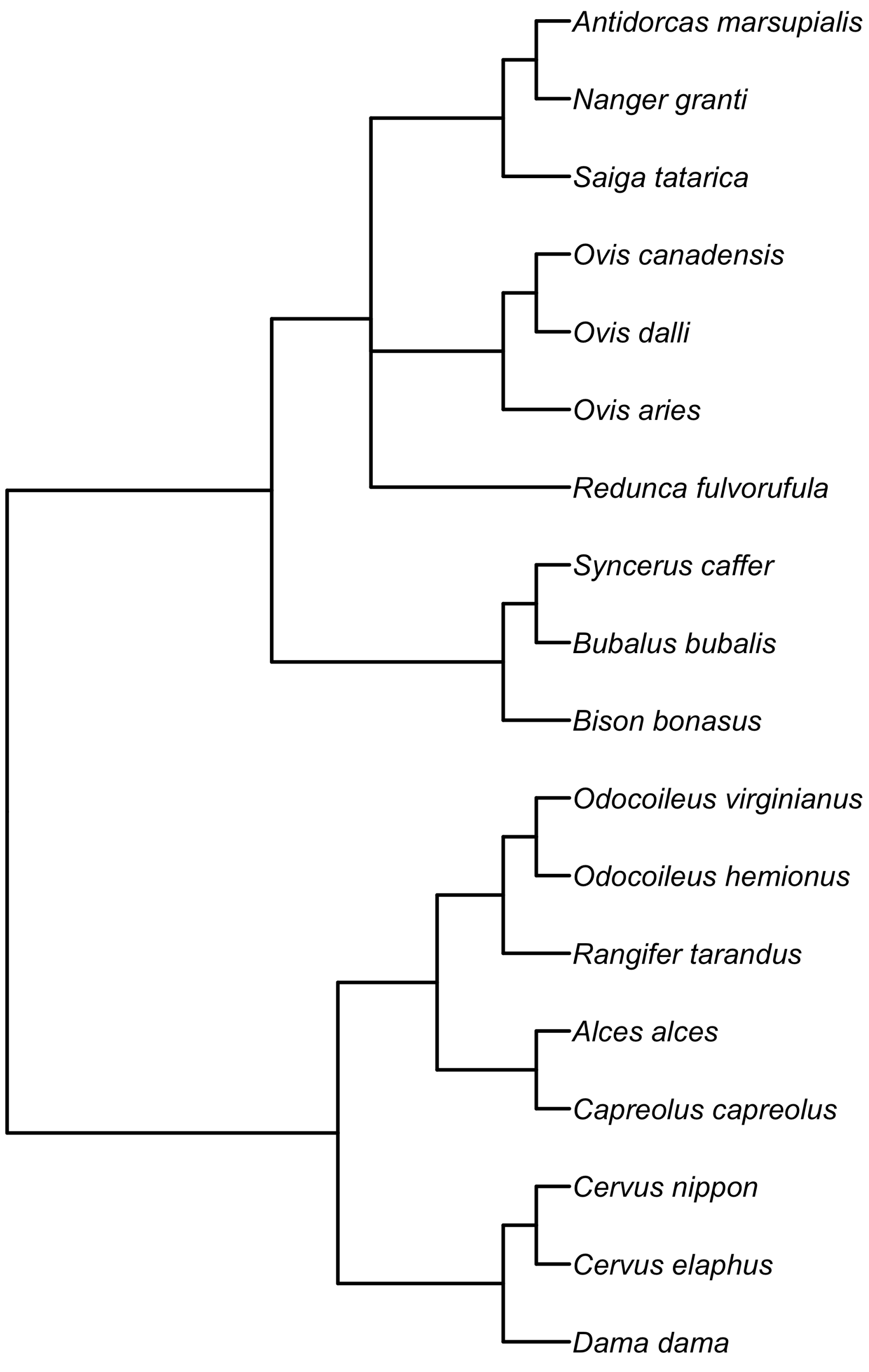

Fig. S2. Phylogeny of ruminant host species included in meta-analysis of helminth parasite effects on free-living ruminant hosts.

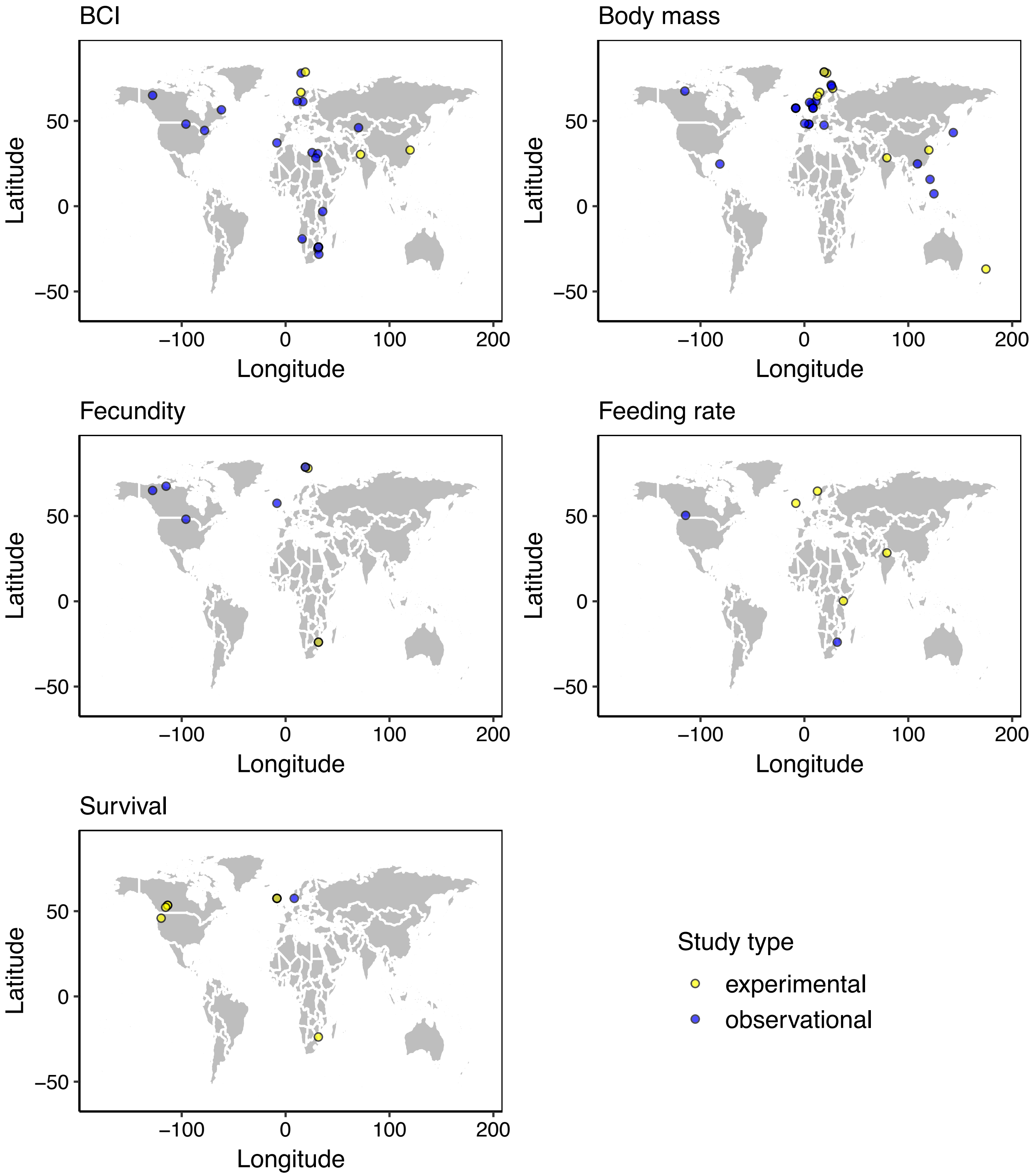

Fig. S3. Locations of studies included in the meta-analysis, mapped separately for each response variable. The final dataset included 259 records from 59 studies spanning 18 host species (Fig. S2) and five global regions: Europe (*n*=27 studies), Africa (*n*=12), North America (*n*=11), Asia (*n*=8), and New Zealand (*n*=1).

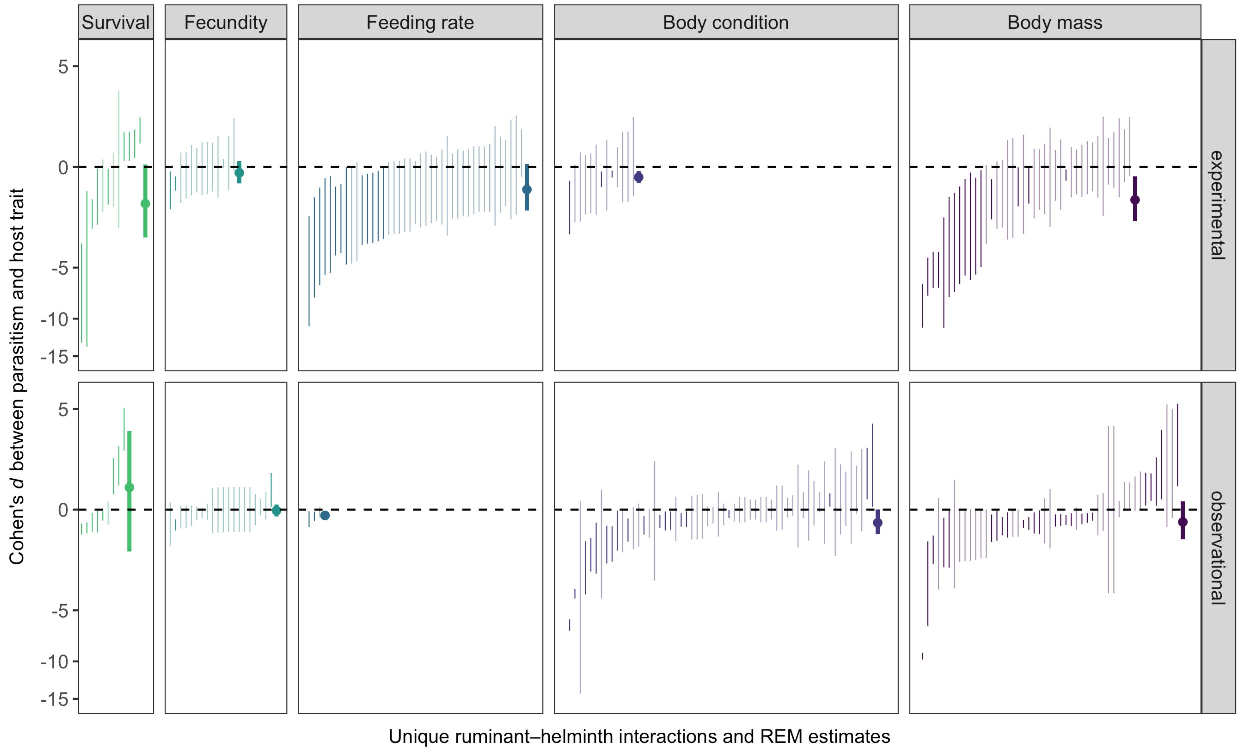

Fig. S4. Meta-analysis results on associations between helminth infection and ruminant host traits. Lines represent the 95% confidence interval for individual effect sizes (Cohen's *d*), shaded by whether they cross zero. Filled circles display the REM mean estimates and 95% confidence intervals. Results are stratified by experimental (*n*=119) and observational (*n*=139) studies. The vertical axes are displayed with a modulus transformation to accommodate skewed effect size distributions.

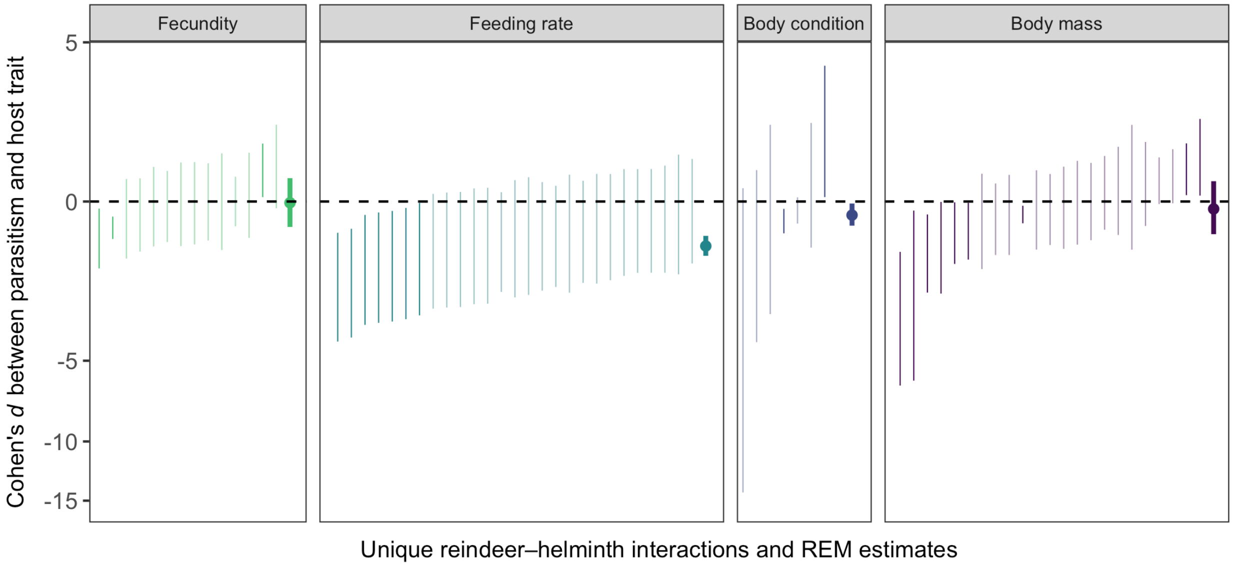

Fig. S5. Meta-analysis results on associations between helminth infection and host traits for reindeer and caribou (both *Rangifer tarandus*). Lines represent the 95% confidence interval for individual effect sizes (Cohen's *d*), shaded by whether they cross zero. Filled circles display the REM mean estimates and 95% confidence intervals. The vertical axes are displayed with a modulus transformation to accommodate skewed effect size distributions.

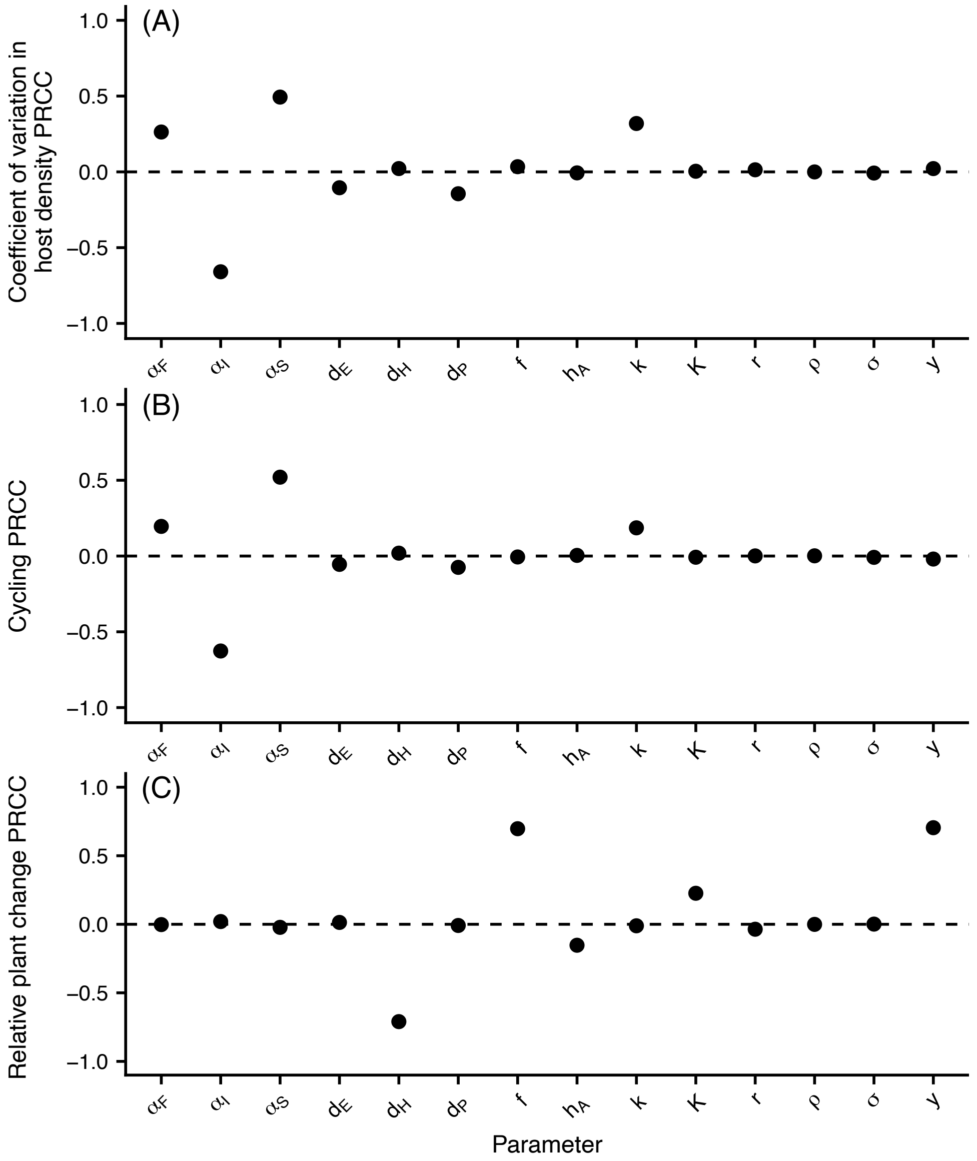

Fig. S6. Global sensitivity analysis of model output to variation in input parameters, as indicated by the partial rank correlation coefficients (PRCC) for the (A) coefficient of variation for host density, a measure of the amplitude of cycles, (B) a binary indicator of the presence of model cycling (0 = no, 1 = yes), and (C) the magnitude of trophic cascades caused by parasitic infection in herbivorous hosts, as estimated by the relative change in primary producer resources between simulations with parasites present and absent. These three measures of cycling were not sensitive to most parameters. However, as shown in (A) and (B), the presence and magnitude of cycling tended to increase with increasing parasite harm to host fecundity, α_F, or host survival, α_S. Increases in the parameter *k*, which indicate decreases in aggregation of adult worms among hosts, also tended to promote cycling. In contrast, only parasite harm to host ingestion, α_I, was strongly stabilizing, as it was the only parameter to exhibit a large negative PRCC with either measure of cycling. Panel (C) shows that stronger trophic cascades tended to occur when hosts exhibited higher feeding rates (f) and fecundity conversion efficiency (y) as well as a lower background death rate (d_H) and a lower half-saturation constant in their type II functional response (h_A). Cascade strength in (C) was not significantly associated with any one type of parasite-induced harm to hosts.

Table S1. Model comparison to select between competing random effects structures in the meta-analysis models. Models were fit using REML with all considered fixed effects (study type and response variable type) and compared with AICc. Models are arranged in order of increasing AICc alongside their random effects structure, number of random effects, the spatial or temporal structure of the covariance matrices, and the estimated fixed effect coefficients.

	**Random effects**				**Fixed effect coefficients from a full model**					
**AICc**	**formula**	**number**	**structure 1**	**structure 2**	**intercept**	**Response: mass**	**Response: fecundity**	**Response: feeding**	**Response: survival**	**study_observational**
135.748821	~1 | study/observation; ~time | trait	2	AR	NA	-0.3579213	0.02242152	0.1673844	0.09587505	0.07786806	0.1338964
135.748821	~1 | study/observation; ~time | trait	2	CAR	NA	-0.3579211	0.02242109	0.16738445	0.09587521	0.07786768	0.13389642
139.564387	~1 | study/observation; ~time | trait	2	CS	NA	-0.3625027	0.03178226	0.16636754	0.08643463	0.08575673	0.13439167
140.131808	~1 | study/observation; ~1 | species; ~1 | phylogeny; ~time | trait	4	AR	NA	-0.357939	0.02243244	0.16738247	0.09587645	0.07788987	0.13389641
140.132872	~1 | study/observation; ~1 | species; ~1 | phylogeny; ~time | trait	4	CAR	NA	-0.3580051	0.02244895	0.16737822	0.09588081	0.07795972	0.13389843
142.839074	~1 | study/observation; ~1 | species; ~1 | phylogeny	4	CS	NA	-0.3610902	0.07474798	0.12612634	0.02722026	0.06939084	0.12065456
142.839383	~1 | study/observation; ~lon + lat | constant	2	SPRAT	NA	-0.3611666	0.07474612	0.12612445	0.02721883	0.0693856	0.12065445
142.840095	~1 | study/observation; ~lon + lat | constant	2	SPEXP	NA	-0.3611588	0.07474796	0.12612602	0.0272203	0.06938886	0.12065187
142.840216	~1 | study/observation; ~lon + lat | constant	2	SPLIN	NA	-0.3610884	0.07474867	0.12612666	0.02721993	0.06939054	0.1206544
142.840287	~1 | study/observation; ~lon + lat | constant	2	SPGAU	NA	-0.361095	0.07474776	0.12612612	0.02721988	0.06939042	0.12065559

142.840856	~1 | study/observation; ~lon + lat | constant	2	SPSPH	NA	-0.3611157	0.07474806	0.12612652	0.02722156	0.06939076	0.12065216
143.947773	~1 | study/observation; ~1 | species; ~1 | phylogeny; ~time | trait	4	CS	NA	-0.362558	0.03182754	0.16636737	0.08643962	0.08582462	0.13438357
144.58664	~1 | study/observation; ~lon + lat | constant; ~1 | species; ~1 | phylogeny; ~time | trait	4	SPGAU	AR	-0.3579457	0.0224124	0.16738121	0.09587538	0.0778698	0.13392691
144.586653	~1 | study/observation; ~lon + lat | constant; ~1 | species; ~1 | phylogeny; ~time | trait	4	SPEXP	AR	-0.357938	0.02244886	0.16738753	0.09588399	0.07789345	0.13389023
144.586695	~1 | study/observation; ~lon + lat | constant; ~1 | species; ~1 | phylogeny; ~time | trait	4	SPSPH	AR	-0.3579245	0.0224114	0.16738295	0.09587314	0.07786768	0.13389811
144.586766	~1 | study/observation; ~lon + lat | constant; ~1 | species; ~1 | phylogeny; ~time | trait	4	SPRAT	AR	-0.3579395	0.02242704	0.16738361	0.09587667	0.07788749	0.13389662
144.586785	~1 | study/observation; ~lon + lat | constant; ~1 | species; ~1 | phylogeny; ~time | trait	4	SPGAU	CAR	-0.3579378	0.02243886	0.16738233	0.09587826	0.07789243	0.1338929
144.587104	~1 | study/observation; ~lon + lat | constant; ~1 | species; ~1 | phylogeny; ~time | trait	4	SPLIN	AR	-0.3579669	0.02243077	0.16738197	0.09587626	0.07789916	0.13389682
144.588532	~1 | study/observation; ~lon + lat | constant; ~1 | species; ~1 | phylogeny; ~time | trait	4	SPEXP	CAR	-0.3580491	0.02244195	0.16737882	0.09587411	0.077941	0.13388543

144.588549	~1 | study/observation; ~lon + lat | constant; ~1 | species; ~1 | phylogeny; ~time | trait	4	SPRAT	CAR	-0.3580832	0.022464	0.16737244	0.09588108	0.0779939	0.13389497
144.588664	~1 | study/observation; ~lon + lat | constant; ~1 | species; ~1 | phylogeny; ~time | trait	4	SPSPH	CAR	-0.3579932	0.02243894	0.16738161	0.09587384	0.0779193	0.13388793
144.58895	~1 | study/observation; ~lon + lat | constant; ~1 | species; ~1 | phylogeny; ~time | trait	4	SPLIN	CAR	-0.3580139	0.02244062	0.16738297	0.09589719	0.07793857	0.13388281
147.222018	~1 | study/observation; ~lon + lat | constant; ~1 | species; ~1 | phylogeny	4	SPGAU	NA	-0.3611153	0.07474824	0.12612596	0.02721951	0.06939044	0.1206539
147.222124	~1 | study/observation; ~lon + lat | constant; ~1 | species; ~1 | phylogeny	4	SPRAT	NA	-0.3611174	0.07474924	0.12612643	0.02721969	0.06939228	0.12065355
147.222225	~1 | study/observation; ~lon + lat | constant; ~1 | species; ~1 | phylogeny	4	SPEXP	NA	-0.3611039	0.07474837	0.12612619	0.02721964	0.0693913	0.1206535
147.222273	~1 | study/observation; ~lon + lat | constant; ~1 | species; ~1 | phylogeny	4	SPSPH	NA	-0.3611024	0.07474881	0.12612618	0.02721913	0.06939192	0.12065285
147.222332	~1 | study/observation; ~lon + lat | constant; ~1 | species; ~1 | phylogeny	4	SPLIN	NA	-0.3611032	0.0747493	0.12612672	0.02722009	0.0693933	0.12065358
148.402833	~1 | study/observation; ~lon + lat | constant; ~1 | species; ~1 | phylogeny; ~time | trait	4	SPLIN	CS	-0.3625328	0.03178334	0.16637151	0.08644194	0.08577287	0.13438661

148.402912	~1 | study/observation; ~lon + lat | constant; ~1 | species; ~1 | phylogeny; ~time | trait	4	SPSPH	CS	-0.3625448	0.03179964	0.16637046	0.08644095	0.08578548	0.13438676
148.403439	~1 | study/observation; ~lon + lat | constant; ~1 | species; ~1 | phylogeny; ~time | trait	4	SPRAT	CS	-0.3625799	0.03182763	0.16636647	0.08644307	0.0858441	0.13438426
148.403459	~1 | study/observation; ~lon + lat | constant; ~1 | species; ~1 | phylogeny; ~time | trait	4	SPEXP	CS	-0.3625903	0.03181147	0.16636788	0.08644203	0.08581422	0.13438851
148.403587	~1 | study/observation; ~lon + lat | constant; ~1 | species; ~1 | phylogeny; ~time | trait	4	SPGAU	CS	-0.3625631	0.03181033	0.16637003	0.0864457	0.08581493	0.1343876

Table S2. Associations between helminth infection and host focal traits from the meta-analysis. Results from the REMs (mean Cohen's *d* and 95% confidence interval) are provided alongside estimates of heterogeneity (I^2) for each host trait, across all data and as stratified by method of inference and for only reindeer and caribou (*Rangifer tarandus*) hosts.

**Response variable**	**Data subset**	***n***	***d***	**95% lower**	**95% upper**	***z***	***p***	I^2
Survival	pooled	21	-0.72	-2.05	0.4	-1.26	0.208	0.99
Fecundity	pooled	33	-0.08	-0.21	0.05	-1.18	0.24	0.29
Feeding rate	pooled	44	-0.48	-1.03	0.03	-1.85	0.065	0.79
BCI	pooled	71	-0.34	-0.66	-0.03	-2.14	0.032	0.95
Body mass	pooled	90	-0.61	-1.11	-0.14	-2.56	0.01	0.57
Survival	experimental	12	-1.2	-2.91	0.06	-0.99	0.32	0.52
Fecundity	experimental	13	-0.15	-0.46	0.15	-1.77	0.077	0
Feeding rate	experimental	41	-0.67	-1.5	0.07	-1.86	0.062	0.91
BCI	experimental	13	-0.28	-0.45	-0.11	-2.61	0.009	0.2
Body mass	experimental	40	-1.05	-2	-0.25	-3.16	0.002	0.2
Survival	observational	9	0.65	-1.42	3.4	-0.31	0.757	0.4
Fecundity	observational	20	-0.02	-0.18	0.13	-2.53	0.012	0.35
Feeding rate	observational	3	-0.15	-0.27	-0.03	0.64	0.523	0.98
BCI	observational	58	-0.36	-0.74	0	-1.19	0.234	0.98
Body mass	observational	49	-0.34	-0.92	0.22	-1.94	0.052	0.95
Fecundity	*Rangifer tarandus*	14	-0.02	-0.45	0.41	-0.09	0.928	0.62
Feeding rate	*Rangifer tarandus*	27	-0.87	-1.1	-0.64	-7.68	0	0
BCI	*Rangifer tarandus*	7	-0.23	-0.43	-0.03	-2.27	0.023	0.1
Body mass	*Rangifer tarandus*	23	-0.12	-0.6	0.35	-0.5	0.617	0.79

Dataset S1 (separate file). Meta-analysis dataset variable names and definitions

Dataset S2 (separate file). Meta-analysis dataset

Dataset S3 (separate file). Citations for all studies included in the meta-analysis, with notation indicating those publications for which additional information was provided by the authors of the study. PaperID corresponds to the identifier in Dataset S2.

SI References

1. RCoreTeam (2017) R: A language and environment for statistical computing. R Foundation for Statistical ComputingVienna, Austria).
2. Lajeunesse MJ (2016) Facilitating systematic reviews, data extraction and meta-analysis with the metagear package for R. *Methods in Ecology and Evolution* 7(3):323-330.
3. Moher D, Liberati A, Tetzlaff J, Altman Da, & The-PRISMA-Group (2009) Preferred reporting items for systematic reviews and meta-analyses: the PRISMA statement (Reprinted from Annals of Internal Medicine). *Phys. Ther* 89:873-880.
