# Supplementary material for: Sublethal effects of parasitism on ruminants can have cascading consequences for ecosystems
Source: Proc Natl Acad Sci U S A. 2022 May 9;119(20):e2117381119. doi: 10.1073/pnas.2117381119 (PMC9171767; doi:10.1073/pnas.2117381119)
Supplement: Supplementary File [file pnas.2117381119.sd06.pdf]

```c
/* file Eco_No_worm.c */
#include <R.h>
#include <math.h>
static double parms[6];

#define r parms[0]
#define K parms[1]
#define ff parms[2]
#define h_A parms[3]
#define yy parms[4]
#define d_H parms[5]



/* initializer */
void initmod(void (* odeparms)(int *, double *))
{
int N=6;
odeparms(&N, parms);
}



/* Derivatives and 2 output variables */
void derivs (int *neq, double *t, double *y, double *ydot,
double *yout, int *ip)
{

double A = fmax(y[0], 1E-6);
double H = fmax(y[1], 1E-6);

ydot[0] = r*(1-A/K)*A - ff*A*H/(h_A + A);
ydot[1] = yy*ff*A*H/(h_A + A) - d_H*H;

}

/* END file Eco_No_worm.c */
```
